# The Synthesis and Photophysical Performance of a Novel Z-Scheme Ho_2_FeSbO_7_/Bi_0.5_Yb_0.5_O_1.5_ Heterojunction Photocatalyst and the Photocatalytic Degradation of Ciprofloxacin Under Visible Light Irradiation

**DOI:** 10.3390/nano15161290

**Published:** 2025-08-21

**Authors:** Jingfei Luan, Anan Liu, Liang Hao, Boyang Liu, Hengchang Zeng

**Affiliations:** 1 School of Physics, Changchun Normal University, Changchun 130032, China; ananliu2001@outlook.com (A.L.); 19845486007@139.com (L.H.); boyangliu152@outlook.com (B.L.); zenghc23@mails.jlu.edu.cn (H.Z.); 2 State Key Laboratory of Pollution Control and Resource Reuse, School of the Environment, Nanjing University, Nanjing 210093, China

**Keywords:** Ho_2_FeSbO_7_/Bi_0.5_Yb_0.5_O_1.5_ heterojunction photocatalyst, ciprofloxacin, visible light irradiation, degradation mechanism

## Abstract

A pyrochlore-type crystal structure photocatalytic nanomaterial, Ho_2_FeSbO_7_, was successfully synthesized using a hydrothermal method. Additionally, a fluorite-structured Bi_0.5_Yb_0.5_O_1.5_ was prepared via rare earth Yb doping. Finally, a novel Ho_2_FeSbO_7_/Bi_0.5_Yb_0.5_O_1.5_ heterojunction photocatalyst (HBHP) was fabricated using a solvothermal method. The crystal structure, surface morphology, and physicochemical properties of the samples were characterized using XRD, a micro-Raman spectrometer, FT-IR, XPS, ultraviolet photoelectron spectroscopy (UPS), TEM, and SEM. The results showed that Ho_2_FeSbO_7_ possessed a pyrochlore-type cubic crystal structure (space group Fd-3m, No. 227), while Bi_0.5_Yb_0.5_O_1.5_ featured a fluorite-type cubic structure (space group Fm-3m, No. 225). The results of the degradation experiment indicated that when HBHP, Ho_2_FeSbO_7_, or Bi_0.5_Yb_0.5_O_1.5_ was employed as a photocatalytic nanomaterial, following 140 min of visible light irradiation, the removal efficiency of ciprofloxacin (CIP) reached 99.82%, 86.15%, or 73.86%, respectively. This finding strongly evidenced the remarkable superiority of HBHP in terms of photocatalytic performance. Compared to the individual catalyst Ho_2_FeSbO_7_, Bi_0.5_Yb_0.5_O_1.5_, or N-doped TiO_2_, the removal efficiency of CIP by HBHP was 1.16 times, 1.36 times, or 2.52 times higher than that by Ho_2_FeSbO_7_, Bi_0.5_Yb_0.5_O_1.5_, or N-doped TiO_2_, respectively. The radical trapping experiments indicated that in the CIP degradation process, the hydroxyl radical owned the strongest oxidation ability, followed by the superoxide anion and the photoinduced hole. These studies are of great significance for the degradation of antibiotics and environmental protection.

## 1. Introduction

In recent years, with the rapid development of the pharmaceutical and medical industries, the use of antibiotics in humans and animals has significantly increased [1,2,3]. The wastewater discharge from antibiotic production enterprises is the main source of antibiotic pollution [4]. Currently, antibiotics have been detected in sewage, surface water, and groundwater worldwide to varying degrees [5,6]. However, once antibiotics enter the environment, they not only disrupt ecological balance and reduce biodiversity but also pose potential threats to human health [7,8,9]. Therefore, it is urgent to develop technical solutions that can effectively remove antibiotic residues from sewage and purify freshwater resources.

Ciprofloxacin (CIP), a synthetic third-generation fluoroquinolone antibiotic with the chemical formula C_17_H_18_FN_3_O_3_, has demonstrated exceptional broad-spectrum bactericidal activity [10,11,12]. When CIP was released into aquatic environments, CIP inhibited microbial growth and reproduction [13]. Due to the bioaccumulative nature of CIP, prolonged exposure significantly increased human morbidity and mortality risks [14,15]. Currently, common treatment methods for removing CIP both domestically and internationally included physical and biological approaches [16,17]. Physical methods mainly relied on complexation, electrostatic adsorption, and van der Waals forces to adsorb CIP on surfaces for removal of CIP [18,19]. Biological methods utilized microbial metabolism for degrading CIP into CO_2_, H_2_O, and other small molecules [20,21]. In addition, other methods such as flocculation sedimentation, Fenton oxidation, and electrochemical oxidation were also available for removing CIP [22,23,24]. However, these methods were either inefficient and lead to increased treatment costs, or these methods generated new pollutants which required additional treatment [25]. Due to all these reasons, these methods cannot be applied in actual CIP pollution control projects.

To address the aforementioned challenges, researchers have been constantly exploring more efficient and environmentally friendly methods to degrade CIP. In recent years, photocatalysis technology has demonstrated great potential in environmental protection and governance due to its advantages of being clean, efficient, and multifunctional, attracting the attention of many researchers [26,27,28]. This technology utilized light energy to excite electron–hole pairs on the surface of the catalyst [29], where electrons in the conduction band could reduce O_2_ to generate highly reactive •O_2_^−^ radicals, while holes in the valence band could oxidize OH^−^ to produce highly reactive •OH radicals [30]. These free radicals could rapidly decompose organic pollutants [31]. In this process, the photocatalyst played a decisive role as the core of the key technology.

Conventional photocatalytic nanomaterials such as TiO_2_ and CuO own limitations such as a weak response to visible light and low photogenerated electron mobility, which significantly restricted the improvement of their photocatalytic performance [32,33]. To more efficiently utilize the visible light portion of solar energy, constructing composite materials has become an effective strategy [34,35,36]. Studies have shown that A_2_B_2_O_7_-type compounds exhibit excellent photocatalytic activity [37,38]. Devi et al. Discovered that the photocatalytic nanomaterial YGdTi_2_O_7_ could achieve a removal efficiency (RME) of 66% for methylene blue after visible light irradiation of 180 min [39]. Additionally, Zhang et al. demonstrated that the La_2_Ce_2_O_7_ photocatalyst achieved a RME of 76.6% for methyl orange after visible light irradiation of 300 min [40]. Meanwhile, fluorite-structured oxides, due to their unique oxygen vacancy defects, tunable band structures, and excellent stability, have shown great potential in the fields of photocatalytic degradation of pollutants, hydrogen production, and CO_2_ reduction [41,42]. For example, Mesut Sezer et al. studied the degradation of bisphenol A using fluorite-structured nano-CeO_2_ materials [43]. Mengqi Jiang et al. explored the optical diagnostic application of the CeO_2_ nanocatalyst for the ammonia combustion process of compression ignition engines [44]. Based on these research results, this study aims to develop a new type of composite photocatalytic nanomaterial by doping with rare earth metals to synergistically optimize light absorption and charge transport properties. The specific details will be further elaborated in the following text.

However, despite the significant advantages of photocatalytic technology, there were still issues such as low quantum efficiency and insufficient utilization of visible light [45]. Therefore, in connection with the core challenges which are commonly encountered in photocatalytic technology, such as high rates of photogenerated charge recombination, insufficient visible light response, easy deactivation of catalysts, and difficulty in precisely controlling reaction selectivity [46,47], it is urgent to develop a novel photocatalyst which features both efficient charge separation and transport, long-term cycling stability, and environmental friendliness and sustainability at the same time [48]. Above novel photocatalyst has become the key for breaking through the current technological bottlenecks and promoting practical applications.

In previous studies, we found a large number of A_2_B_2_O_7_-type pyrochlore-structured materials which exhibited excellent photocatalytic performance in photocatalytic reactions, while fluorite-structured CeO_2_ had also been widely used in the field of photocatalysis [49,50,51]. By replacing rare earth elements and transition metal elements, the Ho_2_FeSbO_7_ catalyst and the Bi_0.5_Yb_0.5_O_1.5_ catalyst were designed and prepared. Among the Ho_2_FeSbO_7_ catalyst and the Bi_0.5_Yb_0.5_O_1.5_ catalyst, the outermost electrons of Ho and Yb were located in the 4f orbital. As a result, the energy which was required for electron transitions in the 4f orbital matched the energy of visible light, and, accordingly, the photocatalytic activity of Ho_2_FeSbO_7_ or i_0.5_Yb_0.5_O_1.5_ could be enhanced by the visible light response capability [52].

In order to further improve the photocatalytic performance of the Ho_2_FeSbO_7_ catalyst or Bi_0.5_Yb_0.5_O_1.5_ catalyst, the following methods such as surface modification, doping modification, deposition of noble metals, and the construction of heterojunctions could be considered [53,54,55]. Among the above methods, the construction of heterojunctions has attracted much attention because it could effectively separate photo-induced electrons and photo-induced holes. As a result, the photocatalytic activity of the heterojunction catalyst was significantly enhanced [56]. Moreover, by constructing heterojunctions, not only the spectral response range could be broadened, but also the stability of the catalyst could be improved; thus, achieving novel heterojunctions would become one of the core strategies to break through the bottleneck of photocatalytic technology [57,58]. For instance, Tharit Lerdwiriyanupap et al. studied BiYO_3_/CeO_2_ composite materials, and the results showed that this heterojunction exhibited significantly higher photocatalytic activity compared to pure BiYO_3_ and CeO_2_ [59]. Similarly, Fan Gongduan et al. achieved a higher degradation efficiency of CIP by constructing a Bi_2_MoO_6_/FeVO_4_ heterojunction, which was superior to the photocatalytic activity of a single catalyst [60].

Through the subsequent experimental analysis, it was determined that the conduction band and the valence band of Ho_2_FeSbO_7_ and Bi_0.5_Yb_0.5_O_1.5_ were arranged in an “interlaced” manner, which met the requirements for constructing a Z-scheme heterojunction. Based on the above research results, we developed a new Z-scheme Ho_2_FeSbO_7_/Bi_0.5_Yb_0.5_O_1.5_ heterojunction photocatalyst (HBHP), which could efficiently remove CIP under visible light irradiation and exhibited excellent photocatalytic performance.

## 2. Experimental Section

### 2.1. Chemicals and Materials

Ho(NO_3_)_3_·6H_2_O (purity 99.99%), Fe(NO_3_)_3_·6H_2_O (purity 99.99%), Bi_2_O_3_ (purity 99.99%), Yb_2_O_3_ (purity 99.99%), and SbCl_5_ (purity of 99.99%) were all purchased from Aladdin Chemical Reagent Co., Ltd. (Shanghai, China). Isoproply alcohol (IPA, C_3_H_8_O, purity 99.7%), ethylenediaminetetraacetic acid (EDTA, C_10_H_16_N_2_O_8_, purity 99.5%), p-benzoquinone (BQ, C_6_H_4_O_2_, purity 99.5%), absolute ethanol (C_2_H_5_OH, purity 99.5%), octanol (C_8_H_18_O, purity 99.5%), methanol (CH_3_OH, purity 99.5%), and CIP (C_17_H_18_FN_3_O_3_, purity 99.5%) were purchased from China National Pharmaceutical Group Chemical Reagent Co., Ltd. (Shanghai, China).

### 2.2. The Synthesis of Ho_2_FeSbO_7_

The Ho_2_FeSbO_7_ catalyst was successfully prepared by the hydrothermal method. The specific steps were as follows: firstly, 10 mL of Ho(NO_3_)_3_·6H_2_O (1 mol/L), 5 mL of Fe(NO_3_)_3_·6H_2_O (1 mol/L), and 5 mL of SbCl_5_ (1 mol/L) solutions were thoroughly mixed to prepare the precursor solution. Then, the precursor solution was transferred into a polytetrafluoroethylene liner, sealed, and placed in a stainless steel autoclave. The reaction medium was composed of a mixture of glycerol and water with a volume ratio of 1:1, and the polyethylene glycol (PEG) was used as the dispersant. The PEG which was used owned a molecular weight of 400, and the amount of PEG accounted for approximately 60% of the autoclave volume. Next, the autoclave, which possessed a volume of 100 mL, was placed in a high-temperature sintering furnace and heated from a room temperature of 25 °C to 250 °C at a rate of 5 °C/min under a pressure of 120 MPa. Ultimately, the autoclave which contained glycerol, water, and PEG was maintained at 250 °C for 960 min. Afterward, it was naturally cooled to room temperature. The obtained product was centrifugally filtered and alternately washed with deionized water and anhydrous ethanol three times to remove residual impurities. Subsequently, the washed material was vacuum-dried at room temperature. Finally, the dried powder was pressed into thin sheets and then crushed into fine particles to obtain the pure-phase Ho_2_FeSbO_7_ catalyst.

### 2.3. The Synthesis of Bi_0.5_Yb_0.5_O_1.5_

The Bi_0.5_Yb_0.5_O_1.5_ catalyst was successfully prepared by high-temperature solid-state sintering. The specific steps were as follows: firstly, Bi_2_O_3_ (purity 99.99%) and Yb_2_O_3_ (purity 99.99%) powders were weighed in a molar ratio of 1:1 and thoroughly ground and mixed for 3 h. Then, the mixed powder was placed in a drying oven and dried at 200 °C for 6 h to remove physically adsorbed water. After that, the dried powder was evenly spread in an alumina crucible and placed in a high-temperature furnace for calcination. The calcination program was set as follows: heating from room temperature (25 °C) at a rate of 5 °C/min to 450 °C, and holding at 450 °C for 4 h; then, heating at a rate of 10 °C/min to 860 °C and holding at 860 °C for 12 h. Finally, the sample was naturally cooled to room temperature with the furnace and taken out, and then thoroughly ground in a mortar to obtain the desired pure-phase Bi_0.5_Yb_0.5_O_1.5_ catalyst.

### 2.4. The Preparation of Ho_2_FeSbO_7_/Bi_0.5_Yb_0.5_O_1.5_ Heterojunction Photocatalyst

HBHP was prepared by the solvothermal method. The specific steps were as follows: firstly, 0.0015 mol of the successfully prepared Ho_2_FeSbO_7_ powder and 0.0015 mol Bi_0.5_Yb_0.5_O_1.5_ powder were taken and uniformly mixed with 300 mL of octanol (C_8_H_18_O). Subsequently, the obtained mixture was transferred to an ultrasonic bath and treated under ultrasonic conditions for 1 h to ensure thorough dispersion. Then, the mixture was heated to 150 °C and stirred continuously at this temperature for 2 h to promote the formation of the Ho_2_FeSbO_7_/Bi_0.5_Yb_0.5_O_1.5_ heterojunction photocatalytic material. After the reaction was completed, the system was naturally cooled to room temperature, and the product was obtained by centrifugal separation. It was then washed multiple times with anhydrous ethanol to remove residual solvents and other impurities. Finally, the purified powder was dried in a vacuum oven at 100 °C for 6 h and stored in a desiccator for future use. Thus, the Ho_2_FeSbO_7_/Bi_0.5_Yb_0.5_O_1.5_ heterojunction photocatalytic material was successfully prepared.

### 2.5. The Preparation of Nitrogen-Doped Titanium Dioxide

N-doped TiO_2_ was synthesized through high-temperature nitrogen treatment. In this experiment, the TiO_2_ precursor used was in the anatase phase. The TiO_2_ powder was placed in a vacuum drying oven and dried at 80 °C for 10 h to eliminate physically absorbed water molecules. Subsequently, 1–2 g of the dried TiO_2_ powder was evenly distributed in a quartz crucible. The crucible containing the sample was positioned in the constant-temperature zone of a tube furnace, and the reaction chamber was sealed. Pure argon gas (flow rate of 200 mL/min) was introduced for 30 min. The temperature increased from room temperature (25 °C) to 500 °C at a heating rate of 5 °C/min over a period of 95 min. Upon reaching the target temperature, the gas flow was switched to a mixture of NH_3_/Ar (NH_3_ at 50 mL/min and Ar at 50 mL/min), and the sample was maintained at this temperature for 3 h. Following the reaction, the ammonia supply was stopped, and pure argon gas (200 mL/min) was reintroduced. The temperature was decreased at a cooling rate of 2 °C/min until it reached below 200 °C, at which point the sample was removed. Finally, the sample was cooled to room temperature under an argon atmosphere, thus completing the preparation of N-doped TiO_2_.

### 2.6. Characterization

The crystal structure of the samples was determined by X-ray powder diffraction (SmartLab, XRD, Rigaku Corporation, Shanghai, China) with a scanning range of 10° to 100°. Chemical bond information was obtained by a WQF-530A Fourier transform infrared spectrometer (FT-IR, Beifang Rayleigh, Beijing, China), with a scanning wavenumber range of 400 cm^−1^ to 4000 cm^−1^. To further analyze the chemical structure and functional group information of the samples, micro-Raman spectroscopy (inVia Reflex, Renisshaw plx, London, UK) was used, with a scanning range of 100 cm^−1^ to 1500 cm^−1^ and a laser wavelength of 532 nm. The optical properties of the catalysts were measured by a UV-3600 ultraviolet-visible diffuse reflectance spectrometer (DRS, Kyoto, Japan) with a measurement range of 200 nm to 800 nm. The chemical valence of the sample surface was analyzed by X-ray photoelectron spectroscopy (PHI 5000 VersaProbe, XPS, UlVAC-PHI, Mito, Japan), with all binding energies calibrated using the C 1s peak (284.8 eV) as a reference. The microstructure and composition of the samples were characterized by transmission electron microscopy (Talos F200X G2, TEM, Thermo Fisher Scientific, Waltham, MA, USA) and scanning electron microscopy (Apreo Chemi, SEM, Thermo Fisher Scientific, Waltham, MA, USA), respectively. The ionization potential of the valence band of the catalysts was determined by ultraviolet photoelectron spectroscopy (Escalab 250 xi, UPS, Thermo Fisher, Waltham, MA, USA) with a He I ultraviolet source (21.2 eV) and an energy resolution of 100 meV. Additionally, the photoluminescence (PL) properties and fluorescence lifetime of the catalysts were measured by a FLS1000 fluorescence spectrometer (Edinburgh Instruments, Edinburgh, UK). The stability of the recyclable practical catalyst was examined using an inductively coupled plasma mass spectrometer (ICP, ICPMS-2040 LF/2050 LF, Shimadzu, Kyoto, Japan).

### 2.7. Photoelectrochemical Measurements

Electrochemical impedance spectroscopy (EIS) was acquired with the assistance of a CHI660D electrochemical station (Chenhua Instruments Co., Ltd., Shanghai, China) with a typical three-electrode configuration. The working electrode (as-fabricated materials), counter electrode (platinum plate), and reference electrode (Ag/AgCl) formed three parts of the above three-electrodes system; concurrently, the electrolyte fluid was a Na_2_SO_4_ watery solution (0.5 mol/L). The light simulation for the experiment was conducted by a 500 W Xe lamp with a 420 nm cutoff filter. The manufacture of the working electrode was as follows: 8 mg of fabricated materials was dispersed into a mixture of 640 μL of ethanol, 960 μL of ultrapure water, and 32 μL nafion solution and sonicated for 50 min. Then, 8 μL of the suspension was taken and evenly dripped on the surface of the polished glass carbon electrode; accordingly, the above suspension which existed on the surface of the glass carbon electrode was put under the infrared light for drying. Eventually, the working electrode was obtained.

### 2.8. Explanation of the Experimental Setup and Procedures

The photochemical reaction test experiments were conducted using a photochemical reaction instrument (CEL-LB70, China Education Jin Guang Technology Co., Ltd., Beijing, China). In the experiments, a 500 W xenon lamp with a cutoff filter (λ ≥ 420 nm) was used as the light source to simulate visible light conditions. The instrument was equipped with 12 quartz tubes, each with a capacity of 45 mL. During each experiment, these 12 quartz tubes were used to load a single reaction solution, with the concentration of the antibiotic CIP set at 0.03 mmol/L and the total reaction volume at 540 mL. The addition amount of the catalytic materials (HBHP, Ho_2_FeSbO_7_, Bi_0.5_Yb_0.5_O_1.5_, and N-doped TiO_2_) was controlled at 0.12 g/L.

Before the light reaction began, the CIP solution containing the catalytic material was placed in a dark environment and stirred magnetically for 45 min to ensure the adsorption–desorption equilibrium between the catalyst, CIP, and the atmosphere was achieved. During the light reaction, 5 mL samples of the CIP solution were taken every 20 min for analysis. After 140 min of light exposure, the catalyst was removed using a 0.22 μm PES polyethersulfone filter membrane, and the remaining CIP concentration was determined using a high-performance liquid chromatograph (HPLC 1260, Agilent Technologies, Santa Clara, CA, USA). A total organic carbon (TOC) analyzer (Sievers 500 RL, Veolia, Brea, CA, USA) was used to assess the degree of mineralization in the CIP degradation experiments. A centrifuge was employed to separate suspended particles in the dispersion system, and potassium hydrogen phthalate (KHP) was used as the TOC calibration sample. The liquid chromatography–mass spectrometry (LC-MS, Thermo Quest LCQ Duo, Thermo Fisher Scientific Corporation, Waltham, MA, USA) was applied to calibrate the intermediate reactants during the calibration experiments. The Beta Basic-C18 HPLC column (150 × 2.1 mm, 5 μm ID, Thermo Fisher Scientific Corporation, Waltham, MA, USA) was applied during the photocatalytic degradation process of CIP. A 20 μL solution, which was obtained after the photocatalytic reaction, was automatically injected into the LC-MS system. The detection wavelength λ_max_ was 277 nm. The mobile phase consisted of 60% methanol and 40% ultrapure water. Simultaneously, the above mobile phase flowed through the system at a rate of 0.2 mL/min. The spray voltage was set at 4500 V, while the capillary temperature and the voltage were maintained at 27 °C and 19.00 V, respectively. The mass-to-charge ratio (*m*/*z*) range for analysis was set from 50 to 500.

The incident photon flux after visible light irradiation was measured by a radiometer to be 4.76 × 10^−6^ Einstein L^−1^·s^−1^. By adjusting the distance between the reactor and the light source, the photon flux can be changed. The photocatalytic quantum efficiency (PHE) was calculated using the following formula:

(1) $$\Phi =R/I_{0}$$

Among above parameters, Φ represented the photochemical efficiency (PHE), *R* was the degradation rate of CIP, and $I_{0}$ was the photon flux.

## 3. Results and Discussion

### 3.1. Analysis of Morphological and Structural Characterization

**Figure 1:**
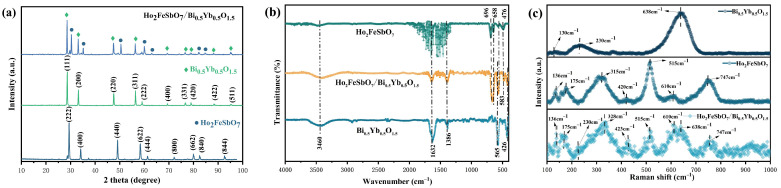
(**a**) XRD spectra, (**b**) FT-IR spectra, and (**c**) Raman spectra of HBHP, Ho_2_FeSbO_7_, and Bi_0.5_Yb_0.5_O_1.5_.

Figure 1 presents the X-ray diffraction (XRD), Fourier transform infrared spectroscopy (FT-IR), and Rama spectra of HBHP, Ho_2_FeSbO_7_, and Bi_0.5_Yb_0.5_O_1.5_. As shown in Figure 1a, the XRD pattern of HBHP contained the main diffraction peaks of Ho_2_FeSbO_7_ and Bi_0.5_Yb_0.5_O_1.5_, indicating that the composite material had been successfully synthesized. Additionally, the characteristic peaks of Fe_2_O_3_, Ho_2_O_3_, or Sb_2_O_5_ were not found in the XRD pattern of Ho_2_FeSbO_7_, suggesting that the catalyst Ho_2_FeSbO_7_ was of high purity and possessed a single-phase structure. Similarly, the diffraction peaks of Bi_2_O_3_ or Yb_2_O_3_ were not detected in the XRD pattern of Bi_0.5_Yb_0.5_O_1.5_, further confirming that Bi_0.5_Yb_0.5_O_1.5_ was a pure single-phase compound. In addition, the XRD pattern of Ho_2_FeSbO_7_, which is shown in Figure S1, was consistent with the standard card of the serpentine structure (PDF#53-1042), while the XRD pattern of Bi_0.5_Yb_0.5_O_1.5_, which is displayed in Figure S2, was in agreement with the standard card of the fluorite-type structure (PDF#04-009-5546).

To further confirm the crystal structure, Rietveld refinement was performed on Ho_2_FeSbO_7_ and Bi_0.5_Yb_0.5_O_1.5_ using Materials Studio software (version 2023). The results are shown in Figures S3a and S4a, respectively. Based on the refined structural parameters, three-dimensional crystal structure models of Ho_2_FeSbO_7_ and Bi_0.5_Yb_0.5_O_1.5_ are constructed and presented in Figures S3b and S4b. The results indicated that Ho_2_FeSbO_7_ owned a pyrochlore-type structure which belonged to the cubic crystal system with the space group Fd-3m (No. 227), and as a result, the lattice constant a of Ho_2_FeSbO_7_ was 10.4632 Å, with an Rp value of 8.41%. The above refinement results indicated a high degree of agreement between the experimental data and the theoretical model. On the other hand, Bi_0.5_Yb_0.5_O_1.5_ exhibited a fluorite structure and also belonged to the cubic crystal system with a space group of Fm-3m (No. 225). As to Bi_0.5_Yb_0.5_O_1.5_, a lattice constant of a = 5.4362 Å was obtained, with an Rp value of 6.34%. The analysis also indicated that Ho_2_FeSbO_7_ and Bi_0.5_Yb_0.5_O_1.5_ possessed good structural compatibility and stability. Tables S1 and S2 list the atomic coordinates and related structural parameters of Ho_2_FeSbO_7_ and Bi_0.5_Yb_0.5_O_1.5_, respectively. In summary, Ho_2_FeSbO_7_ and Bi_0.5_Yb_0.5_O_1.5_ were both high-purity single-phase structures with excellent structural stability, which could provide strong support for their in-depth research in the field of photocatalysis.

The pyrochlore structure was characterized by the chemical formula A_2_B_2_O_6_O’ [61]. Within this arrangement, the A-site metal cation adopted an eight-coordinated hexahedron geometry, while the B-site cation assumed a six-coordinated octahedral configuration [62,63]. An ordered pyrochlore framework was typically formed when the ratio of the radii of the A and B cations fell within the range of 1.46 to 1.78 [64]. In the compound Ho_2_FeSbO_7_, the ionic radii of Ho^3+^, Fe^3+^, or Sb^5+^ was reported as 0.901 Å, 0.550 Å, or 0.600 Å, respectively [65,66]. The resulting ratio lay within the aforementioned range, indicating that Ho_2_FeSbO_7_ adopted a pyrochlore structure, with strong agreement between theoretical predictions and experimental findings. Furthermore, the pyrochlore structure of Ho_2_FeSbO_7_ could be visualized as a three-dimensional network which was formed by Ho^3+^ ions connecting BO_6_ octahedra (where B represented either Fe^3+^ or Sb^5+^) [67,68]. In particular, within the AO_6_O’_2_ sublattice, six Ho-O bonds were measured to be 2.527 Å which was longer than two Ho-O’ bonds which were measured to be 2.243 Å [69]. Additionally, the BO_6_ octahedra (B was Fe^3+^ or Sb^5+^) exhibited structural distortion [70]. Earlier studies had demonstrated that such distortions in crystal geometry could positively influence photocatalytic activity [70]. In the crystal lattice of Ho_2_FeSbO_7_, the B-O-B bond angle was measured to be 130.23°, whereas the Ho-B-Ho bond angle was measured to be 120°. Evidence suggested that the B-O-B bond angle played a crucial role in the delocalization of excited states, with luminescence being enhanced when this angle approached 180° [70]. Moreover, the relatively larger Ho-Fe-O bond angle and Ho-Sb-O bond angle which were presented in Ho_2_FeSbO_7_ further contributed to improved photocatalytic performance. The chemical formula of the fluorite structure was typically expressed as AX_2_ [71,72]. As illustrated in Figure S2b, the anions (O^2−^) adopted a simple cubic arrangement with a coordination number of four. In addition, the metal cations (Bi^3+^ or Yb^3+^) were arranged in a face-centered cubic configuration with a coordination number of eight [73]. Bi_0.5_Yb_0.5_O_1.5_ represented a fluorite-type metal oxide incorporating the rare earth element ytterbium (Yb).

Figure S6 shows the XRD spectrum of N-doped TiO_2_. It could be found from Figure S6 that the N-doped TiO_2_ displayed the characteristic lattice planes of the anatase phase of TiO_2_ (JCPDS No. 21-1272).

Figure 1b presents the Fourier transform infrared (FT-IR) spectra of Ho_2_FeSbO_7_, Bi_0.5_Yb_0.5_O_1.5_, and HBHP. Metal oxide absorption bands typically appeared within the wavenumber range of 100 cm^−1^–1000 cm^−1^ [74]. As shown in Figure 1b, distinct infrared vibration peaks were observed at 426 cm^−1^, 476 cm^−1^, 565 cm^−1^, 583 cm^−1^, 658 cm^−1^, and 696 cm^−1^. As to Bi_0.5_Yb_0.5_O_1.5_, the stretching vibrations of the Bi-O bond and Yb-O bond were identified at 426 cm^−1^ and 565 cm^−1^, respectively [75,76]. In the case of Ho_2_FeSbO_7_, the peak at 476 cm^−1^ corresponded to the bending vibration of the Fe-O bond, while the peak at 583 cm^−1^ was attributed to the absorption peak of Ho-O [77,78]. Furthermore, the stretching vibration and bending vibration of Sb-O linkage and Sb-O-Sb linkage were located at 658 cm^−1^ and 696 cm^−1^, respectively [79,80]. Therefore, the characteristic absorption band which corresponded to Bi-O, Yb-O, Ho-O, Fe-O, Sb-O, or Sb-O-Sb was clearly presented within the 100 cm^−1^–1000 cm^−1^ range. Regarding the spectral region above 1000 cm^−1^, the absorption band at 1386 cm^−1^ could be assigned to the symmetric stretching mode and antisymmetric stretching mode of the C-H bond [81,82]. The peak at 1632 cm^−1^ was associated with the bending vibration of the H-O-H group in chemisorbed water on the catalyst surface [83]. Additionally, the broad band at approximately 3460 cm^−1^ originated from the O-H stretching vibration of adsorbed water molecules [84].

Figure 1c presents the Raman spectra of the Ho_2_FeSbO_7_ sample, Bi_0.5_Yb_0.5_O_1.5_ sample, and HBHP sample. It could be observed from Figure 1c that Bi_0.5_Yb_0.5_O_1.5_ exhibited distinct Raman vibration peaks at 130 cm^−1^, 230 cm^−1^, and 638 cm^−1^. Among them, the peaks at 130 cm^−1^ and 230 cm^−1^ were attributed to the vibration modes of the Bi-O bond, while the peak at 638 cm^−1^ was attributed to the vibration of the Yb-O bond [85,86]. In addition, XRD analysis results confirmed that Ho_2_FeSbO_7_ possessed a pyrochlore structure. According to group theory analysis, this structure could exhibit six Raman active modes within the wavenumber range of 250 cm^−1^–800 cm^−1^, namely, G_Raman_ = A_1g_ + E_g_ + 4F_2g_ [87]. From the spectra in Figure 1c, it could be seen that there were three significant Raman bands in the range of 100 cm^−1^–1000 cm^−1^, which were located at 315 cm^−1^, 515 cm^−1^, and 747 cm^−1^, respectively. The first Raman band (approximately 315 cm^−1^) contained two Raman peaks, which were attributed to the F_2g_ vibration mode and E_g_ vibration mode [88]. Preliminary analysis indicated that the peak at 315 cm^−1^ was mainly caused by the F_2g_ (1) vibration mode, while the peak at 351 cm^−1^ was attributed to the E_g_ vibration mode of the O-sublattice [89]. The Raman peak which was located at 420 cm^−1^ was preliminarily judged to correspond to the F_2g_ (2) Raman active mode of the Ho-O bond [90]. Secondly, the A_1g_ peak which was observed at 515 cm^−1^ was related to the O-B-O bending vibration which was formed by the B-site cations (Fe^3+^ or Sb^5+^) in the BO_6_ octahedron [91,92]. However, the peak position of the wavenumber 515 cm^−1^ was shifted compared to the ideal pyrochlore structure. This might be due to the doping of two different cations (Fe^3+^ or Sb^5+^) at the B-site, which caused structural distortion in the BO_6_ octahedron [93,94]. Additionally, the difference in cation radii also affected the X coordinate of the O(48f) site; thereby, a shift in the wave number was realized [93]. The Raman vibration mode which was observed near 610 cm^−1^ could be attributed to the superposition of A_1g_ + F_2g_ (3) [95]. Finally, the high-frequency Raman peak at 747 cm^−1^ was considered to be closely related to the metal–oxygen stretching vibration mode F_2g_ (4) of the B (Fe^3+^ or Sb^5+^) O_6_ octahedron in the pyrochlore structure [96,97]. For the vibration mode below 300 cm^−1^, it was initially believed that they might result from the anomalous excitation of infrared active mode in Raman spectra. According to the research by Jana et al., the peak at 136 cm^−1^ could be attributed to the infrared active F_1u_ mode, while the signal at 175 cm^−1^ might originate from the second harmonic effect of the A_1g_ phonon mode [98].

In summary, Ho_2_FeSbO_7_ and Bi_0.5_Yb_0.5_O_1.5_ demonstrated superior photocatalytic performance, which was primarily attributed to their distinctive crystal structures and electronic properties. Further investigation into the structural characteristics and electronic characteristics of Ho_2_FeSbO_7_ and Bi_0.5_Yb_0.5_O_1.5_ is crucial for the continued improvement of their photocatalytic efficiency.

**Figure 2:**
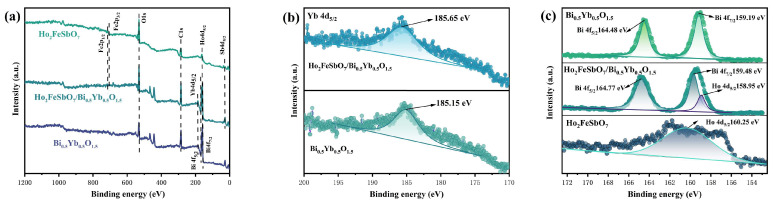

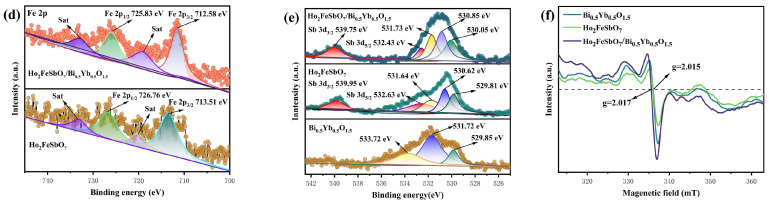
The XPS spectra of the synthesized HBHP, Ho_2_FeSbO_7_, and Bi_0.5_Yb_0.5_O_1.5_: (**a**) survey spectra, (**b**) Yb 3d, (**c**) Bi 4f and Ho 4d, (**d**) Fe 2p, (**e**) Sb 3d and O 1s, and (**f**) EPR spectra.

HBHP, Ho_2_FeSbO_7_, and Bi_0.5Y_b_0.5_O_1.5_ were characterized by X-ray photoelectron spectroscopy (XPS) for analyzing their chemical composition and valence state information of elements. Figure 2 shows the XPS spectrum of HBHP, Ho_2_FeSbO_7_, and Bi_0.5_Yb_0.5_O_1.5_. As shown in Figure 2a, the results indicated that the elements such as Ho, Fe, Sb, Bi, Yb, and O were present in HBHP. Furthermore, by comparing the XPS full spectra of HBHP with those of Ho_2_FeSbO_7_ or Bi_0.5Y_b_0.5_O_1.5_, the corresponding element signal peaks of Ho_2_FeSbO_7_ or Bi_0.5_Yb_0.5_O_1.5_ could be clearly identified in HBHP, indicating that HBHP had been successfully prepared.

As shown in Figure 2b, the Yb 4d_5/2_ peak of Bi_0.5_Yb_0.5_O_1.5_ was displayed with a binding energy of 185.15 eV. However, the binding energy of the Yb 4d_5/2_ peak which belonged to HBHP slightly shifted to 185.65 eV. Similarly, it could be found from Figure 2c that the Bi 4f XPS signal of Bi_0.5_Yb_0.5_O_1.5_ could be observed, with the Bi 4f_7/2_ peak which was located at 159.19 eV and the Bi 4f_5/2_ peak which was located at 164.48 eV. Notably, the Bi 4f_7/2_ peak or the Bi 4f_5/2_ peak which belonged to HBHP showed a slight shift towards higher binding energy, at 159.48 eV (Bi 4f_7/2_) or 164.77 eV (Bi 4f_5/2_) [99]. The spin-orbit splitting value remained at 5.29 eV; thus, the above result indicated that Bi existed within Bi_0.5_Yb_0.5_O_1.5_ or HBHP with the +3 valence state [99]. Additionally, the Ho 4d_5/2_ peak of Ho_2_FeSbO_7_ was located at 160.25 eV, while the Ho 4d_5/2_ peak of HBHP shifted to 158.96 eV [100]. Moreover, in Figure 2d, the XPS peak of Fe 2p in Ho_2_FeSbO_7_ could be clearly observed. As a result, the Fe 2p_1/2_ peak was located at 726.76 eV, while the Fe 2p_3/2_ peak was located at 713.51 eV. Notably, in HBHP, the Fe 2p_1/2_ peak and the Fe 2p_3/2_ peak showed a slight shift towards lower binding energy; specifically, the Fe 2p_1/2_ peak was located at 725.83 eV, while the Fe 2p_3/2_ peak was located at 712.58 eV. The spin-orbit splitting values for both were 13.25 eV, and above result indicated that Fe existed in Ho_2_FeSbO_7_ or HBHP with the +3 valence state [101]. Moreover, the satellite peaks corresponding to the Fe 2p_3/2_ peak and the Fe 2p_1/2_ peak could also be found in Figure 2d, further supporting the above conclusion [101]. In Figure 2e, the Sb 3d_3/2_ peak or the Sb 3d_5/2_ peak within Ho_2_FeSbO_7_ appeared at 539.95 eV or 532.63 eV. While in HBHP, the Sb 3d_3/2_ peak or the Sb 3d_5/2_ peak also shifted towards lower binding energy; namely, the Sb 3d_3/2_ peak was located at 539.75 eV, and simultaneously, the Sb 3d_5/2_ peak was located at 532.43 eV. The spin-orbit splitting value was 7.32 eV, which clearly indicated that Sb existed in Ho_2_FeSbO_7_ or HBHP with the +5 valence state [102].

Figure 2e also presents the deconvolution results of the O 1s spectra for HBHP, Ho_2_FeSbO_7_, and Bi_0.5_Yb_0.5_O_1.5_. According to Figure 2e, the peak which was located at 530.05 eV for HBHP, the peak which was located at 529.81 eV for Ho_2_FeSbO_7_, and the peak which was located at 529.85 eV for Bi_0.5_Yb_0.5_O_1.5_ might be attributed to the lattice oxygen signals [103]. Moreover, the peak which was located at 530.85 eV for HBHP, the peak which was located at 530.62 eV for Ho_2_FeSbO_7_, and the peak which was located at 531.72 eV for Bi_0.5_Yb_0.5_O_1.5_ indicated the presence of oxygen defects in the crystal structure [104]. Particularly, many solid catalysts tend to adsorb moisture or oxygen from the air on their surfaces. Therefore, the peak which was located at 531.73 eV for HBHP, the peak which was located at 531.64 eV for Ho_2_FeSbO_7_, and the peak which was located at 533.72 eV for Bi_0.5_Yb_0.5_O_1.5_ might be attributed to the signals which were caused by surface-adsorbed oxygen [105]. Thus, the position of the O 1s peak within HBHP had shifted significantly compared with that within Ho_2_FeSbO_7_ or Bi_0.5_Yb_0.5_O_1.5_. This change further indicated that there was an interface interaction between Ho_2_FeSbO_7_ and Bi_0.5_Yb_0.5_O_1.5_; concurrently, a strong electronic coupling at the heterojunction interface was realized.

XPS analysis indicated that the Ho, Fe, Sb, Bi, Yb, or O ion within HBHP existed with the oxidation state of +3, +3, +5, +3, +3, or −2. Further analysis of the XPS peaks for Ho_2_FeSbO_7_ and Bi_0.5_Yb_0.5_O_1.5_ revealed that other elements did not exist, which further confirmed that Ho_2_FeSbO_7_ or Bi_0.5_Yb_0.5_O_1.5_ possessed high purity and a single-phase structure.

In addition, we characterized HBHP, Ho_2_FeSbO_7_, and Bi_0.5_Yb_0.5_O_1.5_ by electron paramagnetic resonance (EPR). The results are shown in Figure 2f. An important criterion for determining whether oxygen vacancies existed within a compound was that the g value was close to 2.000 [106]. The g value could be calculated by the following formula:

(2) $$\;g=\frac{h\nu }{\beta B}$$

where h was the Planck constant, v was the photon frequency, g was the dimensionless constant, β was the Bohr magneton, and B was the applied magnetic field [106].

Through calculation and analysis, it was found that the g value of HBHP, Ho_2_FeSbO_7_, or Bi_0.5_Yb_0.5_O_1.5_ was 2.017, 2.015, or 2.015, respectively. Obviously, the g value of 2.017, 2.015, or 2.015 was close to 2.000. The above results indicated that oxygen vacancies existed in HBHP, Ho_2_FeSbO_7_, and Bi_0.5_Yb_0.5_O_1.5_. The above finding was highly consistent with the results of the O 1s peak which was split in XPS data, further verifying the existence of oxygen defects in HBHP, Ho_2_FeSbO_7_, and Bi_0.5_Yb_0.5_O_1.5_.

**Figure 3:**
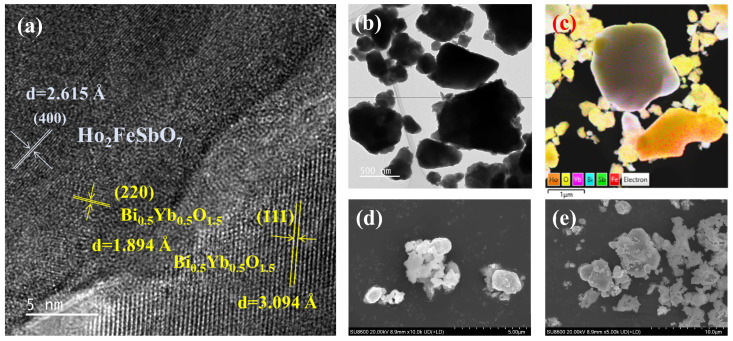
(**a**) HRTEM image of HBHP, (**b**) TEM image of HBHP, (**c**) EDS image of HBHP, (**d**) SEM image of Ho_2_FeSbO_7_, and (**e**) SEM image of Bi_0.5_Yb_0.5_O_1.5_.

Using transmission electron microscopy (TEM), we observed the structural features and intrinsic properties of HBHP at the atomic scale. Meanwhile, in combination with scanning electron microscopy (SEM) and energy dispersive X-ray spectroscopy analysis, we further understood the surface morphology and elemental composition of HBHP. Figure 3a and Figure 3b, respectively, show the high-resolution transmission electron microscopy (HRTEM) image and TEM image of HBHP. From Figure 3b, it could be observed that the small particles were closely surrounding the large particles. Combined with the subsequent EDS characterization, it could be determined that the smaller particles were Ho_2_FeSbO_7_, and the larger particles were the Bi_0.5_Yb_0.5_O_1.5_ catalyst with a fluorite structure. This could further prove the successful synthesis of the heterojunction photocatalyst from a microscopic perspective. In the HRTEM image, the measured interplanar spacing *d* was 2.673 Å, which corresponded to the (400) plane of Ho_2_FeSbO_7_, 1.894 Å which corresponded to the (220) plane of Bi_0.5_Yb_0.5_O_1.5_, or 3.094 Å which corresponded to the (111) plane of Bi_0.5_Yb_0.5_O_1.5_, confirming the coexistence of Ho_2_FeSbO_7_ and Bi_0.5_Yb_0.5_O_1.5_ within HBHP.

**Figure 4:**
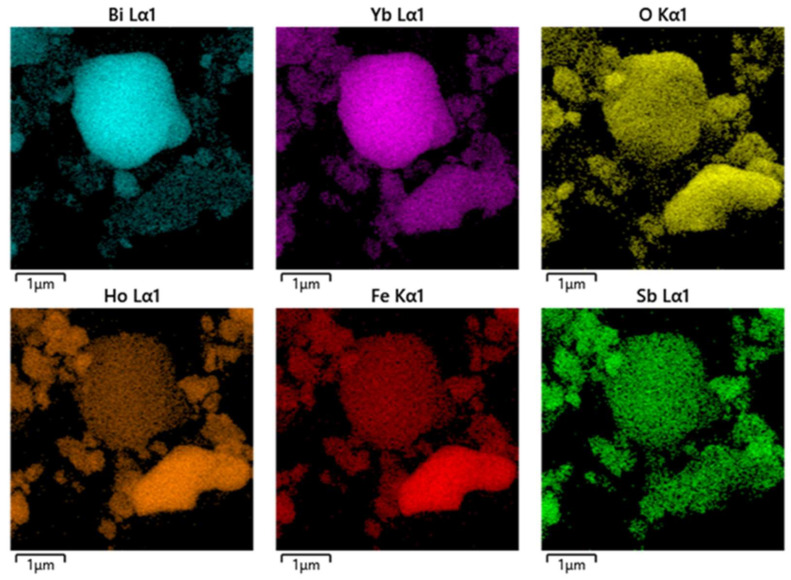
Energy dispersive X-ray spectroscopy elemental mapping of HBHP which originated from the TEM sample (Bi, Yb, and O derived from Bi_0.5_Yb_0.5_O_1.5_, and Ho, Fe, Sb, and O derived from Ho_2_FeSbO_7_).

In addition, Figure 3c shows the EDS image of a certain area of HBHP, and the specific element distribution is presented in Figure 4. Through the element mapping analysis in Figure 4, we further confirmed the presence of the Ho element, Fe element, Sb element, Bi element, Yb element and O element in HBHP. The above results also, once again, indicated that HBHP had been successfully synthesized. These results were also consistent with the XPS data which is shown in Figure 2. In order to gain a more comprehensive understanding of the photophysical properties of the samples, we also conducted SEM morphology observations on a Ho_2_FeSbO_7_ sample and Bi_0.5_Yb_0.5_O_1.5_ sample. Figure 3d shows the SEM image of Ho_2_FeSbO_7_, and Figure 3e presents the SEM image of Bi_0.5_Yb_0.5_O_1.5_. From Figure 3d, it could be observed that the particle contours were clear and the boundaries remained distinct, suggesting that the Ho_2_FeSbO_7_ sample had been well prepared. Additionally, the surface of the Ho_2_FeSbO_7_ particles appeared relatively smooth and flat, without discernible holes, cracks, or significant melting/sintering detected marks, which further verified the successful synthesis of Ho_2_FeSbO_7_. When Figure 3e was examined, the Bi_0.5_Yb_0.5_O_1.5_ particles showed non-uniform dimensions and exhibited a distribution across a size range. These particles which belonged to Bi_0.5_Yb_0.5_O_1.5_ predominantly consisted of irregular polyhedral structures which formed the fundamental building blocks of the Bi_0.5_Yb_0.5_O_1.5_ catalyst system.

Figure S5 shows the EDS spectrum of HBHP. According to Figure S5, the results indicated that the atomic percentages of Bi:Yb:Ho:Sb:Fe:O within HBHP were approximately 1241:1253:446:222:223:6615, which was highly consistent with the XPS results. Based on the results of the above multiple characterization methods, it could be concluded that the high-purity HBHP was successfully synthesized by the solvothermal method.

### 3.2. Optical Characteristics

**Figure 5:**
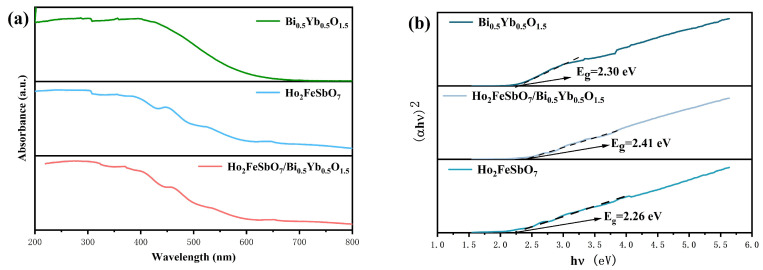
(**a**) The UV-Vis diffuse reflectance spectra of HBHP, Ho_2_FeSbO_7_, and Bi_0.5_Yb_0.5_O_1.5_, and (**b**) the corresponding relationship curves of (*αhv*)^2^ and *hv* for HBHP, Ho_2_FeSbO_7_, and Bi_0.5_Yb_0.5_O_1.5_.

Ultraviolet-visible diffuse reflectance spectroscopy is a technique which utilizes the reflection of light on the surface of a substance for obtaining information about it. For this purpose, we conducted spectral tests on HBHP, Ho_2_FeSbO_7_, and Bi_0.5_Yb_0.5_O_1.5_ under investigation. Thus, the results are shown in Figure 5a. The visible light spectrum ranged from 400 nm to 700 nm. Within the sunlight radiation spectrum, the exposed visible light energy accounted for approximately 43% of the sunlight radiation energy, while the exposed ultraviolet light energy constituted only 5% of the sunlight radiation energy. It could be observed from Figure 5a that the intrinsic absorption edges of all three sample catalysts were within the visible light range, indicating a good response to visible light.

When the incident light irradiated the surface of the nanopowder, diffuse reflection occurred. The Kubelka–Munk transformation was employed as a method to process diffuse reflectance spectroscopy (DRS) data for obtaining absorbance values. Diffuse reflection satisfied the Kubelka–Munk function:

(3) $$F(R_{\infty })=\frac{K}{S}=\frac{{\left(1-R_{\infty }\right)}^{2}}{2R_{\infty }}$$

In the above equation, K represented the absorption coefficient; concurrently, S represented the scattering coefficient; moreover, $R_{\infty }$ denoted the absolute reflectance of an infinite thick sample, and $F(R_{\infty })$ was called the Kubelka–Munk function. Since it was extremely difficult to determine the absolute reflectivity of the catalyst powder, in actual experiments, the relative reflectivity ($r_{\infty }$) was measured using a standard white board BaSO_4_. We assumed that the standard specimen did not exhibit the absorption ability across the investigative spectral range, and, accordingly, its $R_{\infty }$ was effectively equivalent to 1. Under this condition, the relative reflectance of the sample was $r_{\infty }$, where $r_{\infty }\;$=${\;R}_{\infty }$(sample)/$R_{\infty }$(standard). The absorbance data could be obtained via the transformation $A=log(\frac{1}{r_{\infty }})$. Here, *A* represents the apparent absorbance.

In addition, we adopted the formula which was proposed by Davis and Mott et al. for estimating the band gap energy (*E_g_*) of the photocatalysts. The expression of the Tauc function was as follows [107]:

(4) $${\;(ahv)}^{\frac{1}{n}}=A(hv-E_{g})\;$$

In above mathematical equation, *a* represented the absorbance, h was the Planck constant, v was the photon frequency, *A* was the proportionality coefficient, and $E_{g}$ represented the band gap width of the photocatalyst [107]. The test results are shown in Figure 5b. The *E_g_* value of Ho_2_FeSbO_7_, Bi_0.5_Yb_0.5_O_1.5_, or HBHP was 2.26 eV, 2.30 eV, or 2.41 eV, respectively. When the incident photon energy (*hν*) was equal to or greater than the band gap energy (*E_g_*), the photon was absorbed. This absorption excited an electron from the valence band of the photocatalyst to the conduction band of the photocatalyst. As a result, the photo-induced electrons and the photo-induced holes were generated. The *E_g_* which was required for a semiconductor catalyst to absorb visible light energy at 420 nm was calculated to be 2.95 eV. Therefore, the semiconductor catalyst required an *E_g_* value which should be less than 2.95 eV for absorbing visible light energy. The *E_g_* value of Ho_2_FeSbO_7_, Bi_0.5_Yb_0.5_O_1.5_, or HBHP was below 2.95 eV. Accordingly, the above result demonstrated that Ho_2_FeSbO_7_, Bi_0.5_Yb_0.5_O_1.5_, and HBHP possessed visible light response capability. The type of electron transition in the photocatalyst determined the size of the *n* value. The analysis results showed that the *n* value of Ho_2_FeSbO_7_, Bi_0.5_Yb_0.5_O_1.5_, or HBHP was 0.5, indicating that Ho_2_FeSbO_7_, Bi_0.5_Yb_0.5_O_1.5_, or HBHP owned direct band gap transition characteristics [108]. The band gap width of the HBHP composite was larger compared with that of Ho_2_FeSbO_7_ or Bi_0.5_Yb_0.5_O_1.5_. The reason was primarily attributed to the interfacial effects within the heterostructure of HBHP. Charge transfer at the interface induced band bending, specifically at the conduction band minimum of Ho_2_FeSbO_7_ and at the valence band maximum of Bi_0.5_Yb_0.5_O_1.5_, generating a built-in electric field in the interfacial region. This process led to the renormalization of the overall band structure of HBHP, resulting in an increased effective band gap energy of 2.41 eV. This incremental band gap width remained significantly lower than the visible light absorption threshold of 2.95 eV; thus, according to Figure 5a, HBHP maintained good responsiveness under the condition of visible light irradiation. Furthermore, HBHP exhibited a larger specific surface area of 3.09 m^2^/g than its component Ho_2_FeSbO_7_ (2.54 m^2^/g) or Bi_0.5_Yb_0.5_O_1.5_ (2.36 m^2^/g). The increased specific surface area signified a greater heterointerface, which facilitated more extensive contact regions between Ho_2_FeSbO_7_ and Bi_0.5_Yb_0.5_O_1.5_ and provided more active sites.

Figure S7 presents the UV-Vis diffuse reflectance spectra of N-doped TiO_2_. According to Figure S7, the band gap energy *E_g_* value of N-doped TiO_2_ was calculated to be 2.82 eV.

**Figure 6:**
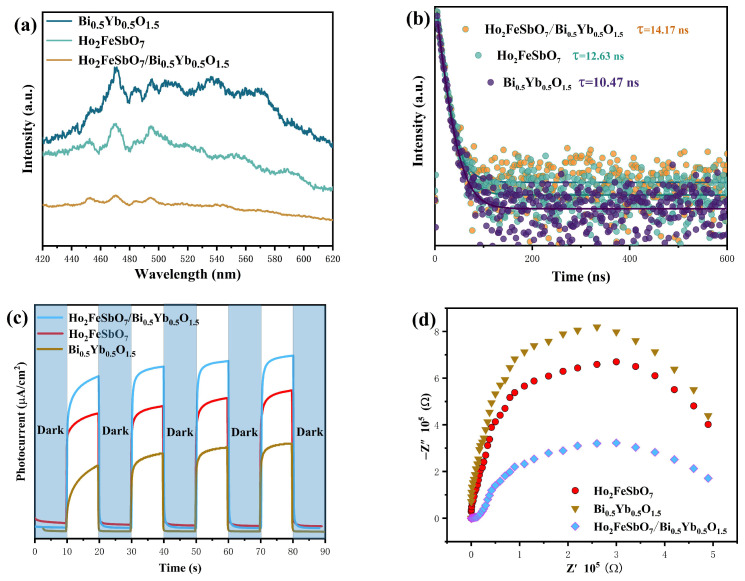
(**a**) PL spectra, (**b**) TRPL spectra, (**c**) transient photocurrent spectrograms, and (**d**) EIS plots of HBHP, Ho_2_FeSbO_7_, and Bi_0.5_Yb_0.5_O_1.5_.

In order to further understand the carrier and the recombination efficiency of the photo-induced electrons and the photo-induced holes, PL spectra and TRPL spectra were used for characterization. In the PL testing experiment, the test was conducted using an excitation wavelength of 340 nm. The results are shown in Figure 6a. Furthermore, in the TRPL test experiment, the excitation wavelength which was used was 340 nm. The emission wavelength of HBHP, Ho_2_FeSbO_7_, or Bi_0.5_Yb_0.5_O_1.5_ was 709 nm, 630 nm, or 601 nm, respectively. The TRPL results are shown in Figure 6b. During the catalytic process, a higher PL intensity indicated a faster recombination rate of the photo-induced electrons and the photo-induced holes, which in turn suggested a lower catalytic effect. As shown in Figure 6a, HBHP exhibited the weakest radiation intensity, indicating that the recombination process of the photo-induced electrons and the photo-induced holes was significantly suppressed. In contrast, the Ho_2_FeSbO_7_ catalyst and Bi_0.5_Yb_0.5_O_1.5_ catalyst showed stronger radiation intensities. The above results suggested that HBHP possessed the lowest recombination efficiency of the photo-induced electrons and the photo-induced holes; thereby, HBHP demonstrated superior catalytic performance during the photocatalytic degradation of CIP.

Figure 6b presents the TRPL spectra of HBHP, Ho_2_FeSbO_7_, and Bi_0.5_Yb_0.5_O_1.5_. As shown in Figure 6b, a double-exponential decay model was used for fitting. The specific fitting equation was as follows [109]:

(5) $$\;I(t)=I(0)+A_{1}exp(-t/\tau_{1})+A_{2}exp(-t/\tau_{2})$$

In the above decay equation, $\tau_{1}$ and $\tau_{2}$, respectively, represented two different recombination mechanisms:$\;\tau_{1}$ possessed a shorter lifetime and mainly resulted from non-radiative recombination at the surface or interface, while $\tau_{2}$ owned a longer lifetime and mainly stemmed from the recombination process within the bulk, including radiative recombination and non-radiative recombination which were caused by bulk defects [110]. The overall average fluorescence lifetime *τ_ave_* could be calculated by the following formula [111]:

(6) $$\;\tau_{ave}=\frac{A_{1}\tau_{1}^{2}+A_{2}\tau_{2}^{2}}{A_{1}\tau_{1}+A_{2}\tau_{2}}$$

It could be found from Figure 6b that the fluorescence lifetime of HBHP ($\tau_{ave}$ = 14.17 ns) was significantly higher than that of Ho_2_FeSbO_7_ ($\tau_{ave}$ = 12.63 ns) and that of Bi_0.5_Yb_0.5_O_1.5_ ($\tau_{ave}$ = 10.47 ns). The above phenomenon indicated that HBHP possessed superior photoelectric properties, a higher photoluminescence quantum yield, and stronger charge transfer capability; thereby, HBHP provided kinetic-level theoretical support for its outstanding photocatalytic performance.

Figure 6c shows the transient photocurrent density of the HBHP, Ho_2_FeSbO_7_ photocatalyst, and Bi_0.5_Yb_0.5_O_1.5_ photocatalyst under the condition of alternating light and dark cycles, reflecting the carrier migration rate [112]. The bias potential for transient photocurrent measurement needed to be set based on the material type, the target reaction, and the stability of the system. In this experiment, we determined the optimal value by optimizing through potential scanning starting from 0 V vs. RHE. The bias potentials for HBHP, Ho_2_FeSbO_7_, and Bi_0.5_Yb_0.5_O_1.5_ were 0.43 V, 0.62 V, and −0.35 V, respectively. According to Figure 6c, the photocurrent density of HBHP was higher than that of Ho_2_FeSbO_7_ or Bi_0.5_Yb_0.5_O_1.5_, indicating that HBHP could generate more effective electrons under the same light conditions and possessed a stronger photoresponse ability. Concurrently, the higher photocurrent density also suggested that HBHP could more effectively separate photo-induced electrons and photo-induced holes during the photocatalytic process. Electrochemical impedance spectroscopy (EIS) could reflect the charge transfer situation between the surface of the photocatalyst and the electrolyte interface [113]. Figure 6d shows the EIS curves of HBHP, Ho_2_FeSbO_7_, and Bi_0.5_Yb_0.5_O_1.5_. The diameter of the semicircle in the mid-frequency region of the Nyquist plot represented Rct. The smaller the Rct was, the lower the charge transfer resistance was, the better the conductivity was, and the faster the catalytic reaction rate was. From Figure 6d, it could be observed that the curves of HBHP, Ho_2_FeSbO_7_, and Bi_0.5_Yb_0.5_O_1.5_ in the mid-frequency region were semicircular, indicating that charge transfer occurred during the photocatalytic process. Rs was 0.002 × 10^5^ Ω. From Figure 6a, the Rct value of HBHP, Ho_2_FeSbO_7_, or Bi_0.5_Yb_0.5_O_1.5_ was 6.45 × 10^5^ Ω, 12.80 × 10^5^ Ω, or 16.38 × 10^5^ Ω, respectively. The results indicated that the charge transfer resistance (Rct) followed the order of Bi_0.5_Yb_0.5_O_1.5_ > Ho_2_FeSbO_7_ > HBHP. Bi_0.5_Yb_0.5_O_1.5_ exhibited the largest Rct value, while HBHP displayed the smallest Rct value. Compared with Bi_0.5_Yb_0.5_O_1.5_ or Ho_2_FeSbO_7_, HBHP possessed the smallest Rct value, and as a result, HBHP could display a faster catalytic reaction capability. HBHP possessed the smallest Rct value, demonstrating that HBHP owned a greater reaction rate than Ho_2_FeSbO_7_ or Bi_0.5_Yb_0.5_O_1.5_. This further implied that HBHP exhibited a higher photocatalytic performance. In the EIS testing experiments, the bias potential which was employed for HBHP, Ho_2_FeSbO_7_, or Bi_0.5_Yb_0.5_O_1.5_ was 0.43 V, 0.62 V, and −0.35 V, respectively.

Based on the above comprehensive experimental results of PL, TRPL, EIS and transient photocurrent density, HBHP demonstrated outstanding performance in multiple key performance indicators, fully indicating that the photocatalytic performance of HBHP was superior to that of Ho_2_FeSbO_7_ or Bi_0.5_Yb_0.5_O_1.5_.

### 3.3. Evaluation of Photocatalytic Activity

**Figure 7:**
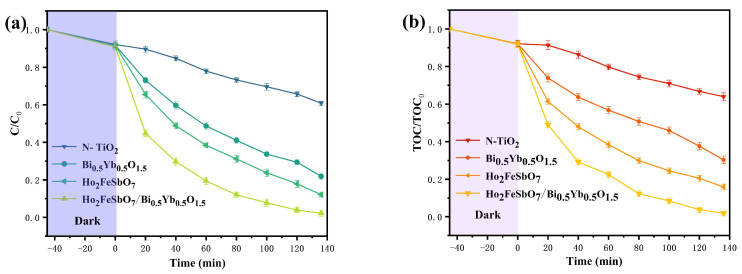
(**a**) The variation curve of CIP concentration with time and (**b**) the variation curve of TOC concentration with time during the degradation of CIP by using HBHP, Ho_2_FeSbO_7_, Bi_0.5_Yb_0.5_O_1.5_, or N-doped TiO_2_.

Figure 7a presents the variation curves of CIP density during the degradation process using HBHP, Ho_2_FeSbO_7_, Bi_0.5_Yb_0.5_O_1.5_, or N-doped TiO_2_. After dark adsorption for 45 min, the CIP adsorption removal rate had reached 6.5%. Dark adsorption referred to the process of adsorbing CIP onto the catalyst surface in a dark environment without its degradation. Under the condition of visible light irradiation, it could be observed that the CIP concentration had significantly decreased. The removal rate of CIP could be calculated by the formula $(1-C/C_{o})\times 100\%$, where *C* represented the instantaneous saturation concentration and *C_o_* represented the initial saturation concentration in this mathematical formula. In accordance with Figure 7a, the results showed that after visible light irradiation of 140 min, the reaction rate of CIP removal within the solution by HBHP was 3.69 × 10^−9^ mol·L^−1^·s^−1^, and the PHE was 0.07758; simultaneously, the (RME) was as high as 99.82%. Similarly, when Ho_2_FeSbO_7_ was utilized as the catalyst to remove CIP from the solution, the RME was 86.15%, and the reaction rate was 3.17 × 10^−9^ mol·L^−1^·s^−1^; concurrently, the PHE was 0.0665%. When Bi_0.5_Yb_0.5_O_1.5_ was used as the catalyst for removing CIP from the solution, the RME was 73.86%, and the reaction rate was 2.69 × 10^−9^ mol·L^−1^·s^−1^; simultaneously, the PHE was 0.0565%. However, when N-doped TiO_2_ was utilized as the catalyst for removing CIP from the solution, the RME was only 39.56%, and the reaction rate was 1.55 × 10^−9^ mol·L^−1^·s^−1^; meanwhile, the PHE was 0.0325%. The above results showed that the photocatalytic degradation efficiency of CIP using HBHP was the highest, followed by that with Ho_2_FeSbO_7_, Bi_0.5_Yb_0.5_O_1.5_, or N-doped TiO_2_ as a catalyst in sequence. Compared with the RME of the other three catalysts, the RME of HBHP was 1.16 times that of Ho_2_FeSbO_7_, 1.35 times that of Bi_0.5_Yb_0.5_O_1.5_, and 2.52 times that of N-doped TiO_2_, respectively.

Figure 7b shows the variation curve of TOC concentration via time during the degradation of CIP with HBHP, Ho_2_FeSbO_7_, Bi_0.5_Yb_0.5_O_1.5_, or N-doped TiO_2_ as catalyst. The RME of *TOC* concentration could be calculated by the formula $(1-TOC/{TOC}_{o})\times 100\%$, where *TOC* represented the instantaneous total organic carbon concentration and *TOC_o_* represented the initial total organic carbon concentration. According to Figure 7b, the results indicated that the saturation concentration of TOC gradually decreased under continuous visible light irradiation. After visible light irradiation of 140 min, the RME of TOC concentration by HBHP reached 97.63%; additionally, the RME of TOC concentration by Ho_2_FeSbO_7_, Bi_0.5_Yb_0.5_O_1.5_, or N-doped TiO_2_ was 81.62%, 67.86%, or 33.45%, respectively. The above results indicated that HBHP possessed the highest mineralization effect on TOC compared with Ho_2_FeSbO_7_, Bi_0.5_Yb_0.5_O_1.5_, or N-doped TiO_2_. Compared with the RME of the TOC concentration using the other three catalysts, the RME of the TOC concentration using HBHP was 1.19 times that using Ho_2_FeSbO_7_, 1.43 times that using Bi_0.5_Yb_0.5_O_1.5_, and 2.91 times that using N-doped TiO_2_.

In summary, compared with Ho_2_FeSbO_7_, Bi_0.5_Yb_0.5_O_1.5_, and N-doped TiO_2_, HBHP demonstrated superior catalytic performance in both CIP removal process and TOC removal process. The above result was consistent with the previous analysis and further validated the high efficiency of HBHP in antibiotic degradation.

**Figure 8:**
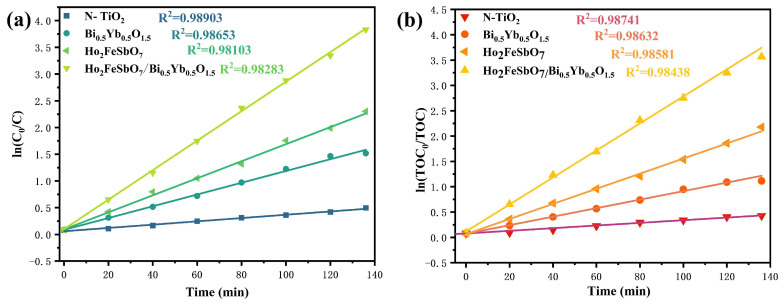
(**a**) CIP concentration degradation kinetics curve and (**b**) TOC concentration degradation kinetics curve in the first order under visible light irradiation by using HBHP, Ho_2_FeSbO_7_, Bi_0.5_Yb_0.5_O_1.5_, or N-doped TiO_2_.

Figure 8a presents the first-order kinetic curves of CIP degradation with HBHP, Ho_2_FeSbO_7_, Bi_0.5_Yb_0.5_O_1.5_, or N-doped TiO_2_ as catalyst. The kinetic constants were calculated based on the equation $ln(C_{o}/C)=K_{C}t$, where *C_o_* was the initial concentration and *C* was the instantaneous concentration. It could be found from Figure 8a that the reaction rate constant during CIP degradation by HBHP was 0.02413 min^−1^, which was significantly higher than that by Ho_2_FeSbO_7_ (0.01203 min^−1^), that by Bi_0.5_Yb_0.5_O_1.5_ (0.00869 min^−1^), and that by N-doped TiO_2_ (0.00285 min^−1^), indicating that HBHP owned excellent photocatalytic performance in CIP degradation compared with Ho_2_FeSbO_7_, Bi_0.5_Yb_0.5_O_1.5_, or N-doped TiO_2_. Figure 8b presents the first-order kinetics of *TOC* concentration variation for CIP degradation using HBHP, Ho_2_FeSbO_7_, Bi_0.5_Yb_0.5_O_1.5_, or N-doped TiO_2_. The first-order kinetic constant could be calculated by the formula $ln({TOC}_{o}/TOC)=K_{TOC}t$. In the above equation, *TOC* represented the instantaneous saturation concentration, and *TOC_o_* represented the initial saturation concentration. It could be revealed from Figure 8b that the kinetic constant for TOC concentration variation for CIP degradation using HBHP was 0.02086 min^−1^, while the kinetic constant for TOC concentration variation for CIP degradation with Ho_2_FeSbO_7_, Bi_0.5_Yb_0.5_O_1.5_, or N-doped TiO_2_ as a catalyst was 0.01086 min^−1^, 0.00661 min^−1^, or 0.00243 min^−1^, respectively. Notably, the *k_TOC_* value of HBHP, Ho_2_FeSbO_7_, Bi_0.5_Yb_0.5_O_1.5_, or N-doped TiO_2_ was lower than the *k_C_* value of HBHP, Ho_2_FeSbO_7_, Bi_0.5_Yb_0.5_O_1.5_, or N-doped TiO_2_, indicating the formation and accumulation of intermediate products during the photodegradation process of CIP. The above results once again demonstrated that HBHP possessed excellent photocatalytic performance for TOC mineralization during the degradation process of CIP.

Furthermore, Table S3 shows the impact of different catalyst amounts of HBHP on the removal efficiency of CIP. The results showed that when the dosage of HBHP was 0.12 g/L, the removal rate of CIP was the highest.

In order to simulate true industrial wastewater environment and different water sources, we added ultrapure water which was supplemented with various anions to the photocatalytic reaction system. Experimental studies were conducted to evaluate the impact of the time of visible light irradiation on the degradation efficiency of CIP. Figures S10 and S11 display the influence of different anions on the degradation removal efficiency of CIP using HBHP under visible light irradiation. It could be found from Figure S10 that the concentration of CIP gradually decreased when the time of visible light irradiation increased. Impressively, after visible light exposure of 140 min, the removal rate of CIP was 100% using HBHP. Additionally, in the photocatalytic reaction system using HBHP, introducing 10 mmol/L NO_3_^−^ resulted in a removal rate of 96.2% for CIP after visible light irradiation of 140 min. Similarly, in the photocatalytic reaction system using HBHP, adding 10 mmol/L SO_4_^2−^ led to a removal rate of 98.4% for CIP after visible light irradiation of 140 min. In contrast, after visible light exposure of 140 min using HBHP, adding 10 mmol/L CO_3_^2−^ or 10 mmol/L Cl^−^ to the photocatalytic reaction system resulted in a CIP removal rate of 56.2% or 50.0%, respectively. These findings emphasized the significant impact of Cl^−^ ions which existed in the water source on the degradation efficiency of CIP. A higher proportion of Cl^−^ led to a significant consumption of •OH and photogenerated holes; ultimately, the availability of free radicals was reduced and the photodegradation procedure was hindered. The inhibitory effect of NO_3_^−^ or SO_4_^2−^ on the photodegradation removal efficiency of CIP was relatively small. This was because NO_3_^−^ or SO_4_^2−^ only consumed the photo-induced holes; thus, a milder inhibitory effect was realized.

Figure S12 demonstrated the influence of different pH values on the removal efficiency of CIP using HBHP under the condition of visible light irradiation. Notably, according to Figure S12, after the dark adsorption of 45 min, the removal efficiency of CIP using HBHP decreased significantly under acidic condition (pH value < 7) during the subsequent visible light irradiation. Excessively strong acidity could reduce or inhibit the generation of active species such as hydroxyl radical (•OH), as a result, the degradation efficiency of CIP was lowered. Furthermore, under the strongly alkaline condition (pH value > 7), the removal efficiency of CIP also decreased markedly. The above results were realized because the deprotonation of the carboxyl groups which belonged to CIP rendered the negative charge on the surface of CIP; accordingly, the electrostatic repulsion was created owing to the existence and inter-reaction of the negative charge which appeared on the surface of CIP and the photo-induced electrons which appeared on the surface of HBHP. Thereby, the effect of the electrostatic repulsion could reduce the adsorption efficiency of CIP, and as a result, the degradation efficiency of CIP was decreased. Under neutral condition (pH value = 7), CIP existed in its zwitterionic form, indicating that the charge amount which appeared on the surface of CIP would be equal to zero; accordingly, the above result minimized electrostatic repulsion which occurred between the surface of HBHP and the surface of CIP, and as a result, the surface of HBHP could easily adsorb the CIP; ultimately, the degradation efficiency of CIP was promoted. In conclusion, the optimal pH value for degrading CIP using HBHP was 7. This conclusion can be clearly observed in Figure S12.

The effect of CIP concentration on the degradation efficiency of CIP was investigated using a HBHP dosage of 0.12 g/L at a pH value of 7. As shown in Figure S13, the CIP solution concentrations which ranged from 0.01 mmol/L to 0.05 mmol/L were evaluated. At a CIP concentration of 0.01 mmol/L or 0.02 mmol/L, the degradation process was nearly completed within 80 min. Furthermore, when the CIP concentration increased, the degradation rate of CIP gradually decreased. The above reduction occurred because excess CIP obstructed the incident light; subsequently, the utilization efficiency of light energy was diminished, and simultaneously, the amount of the active sites on the surface of HBHP decreased; accordingly, the degradation rate of CIP was decreased.

**Figure 9:**
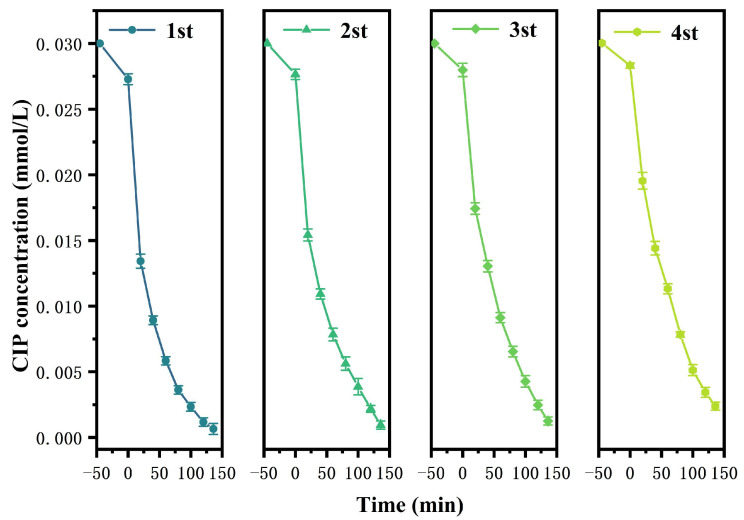
The variational cycle curves of CIP concentration during the photocatalytic degradation process by using HBHP.

**Figure 10:**
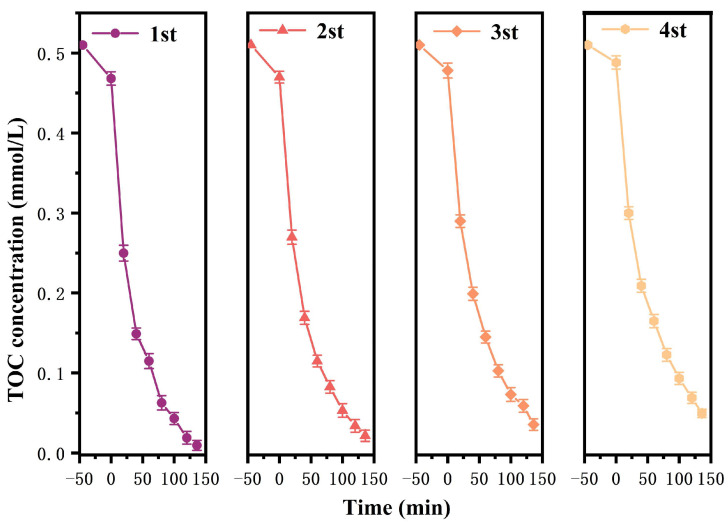
The variational cycle curves of TOC concentration during the photocatalytic degradation process of CIP by using HBHP.

Figure 9 shows the removal efficiency change curve of the repeated degradation for CIP using HBHP under visible light irradiation. It could be seen from Figure 9 that after light exposure of 140 min, the RME of CIP using HBHP in the first cycle reached 99.82%, and in rapid sequence, after four experimental cycles, the RME of CIP using HBHP remained at 93.53%, indicating that HBHP possessed high stability and continuous degradation ability. In addition, Figure 10 presents the variational trend cycle curves of TOC concentration during the photocatalytic degradation process of CIP using HBHP under the same experimental conditions. After conducting one cycle of the degradation experiment, the catalyst was removed from the solution by centrifugation. As shown in Figure 10, the results showed that after four cycles of experiments, the RME of TOC concentration was still as high as 90.34%, demonstrating that HBHP owned excellent mineralization performance. In this experiment, XRD and SEM tests were conducted on HBHP which was obtained after the cyclic degradation of CIP. The experimental results are shown in Figures S8 and S9. From Figure S8, it could be concluded that the HBHP structure remained stable after the cyclic degradation process. Additionally, After the cyclic degradation experiment was completed, the recovered HBHP was subjected to mass spectrometry analysis using inductively coupled plasma (ICP). The results showed that the HBHP structure was stable and that metal leaching did not occur.

In summary, HBHP not only possessed excellent catalytic activity and mineralization ability, but also maintained stability during multiple experimental cycles of utilization; thus, HBHP revealed broad prospects in the field of photocatalytic applications.

**Figure 11:**
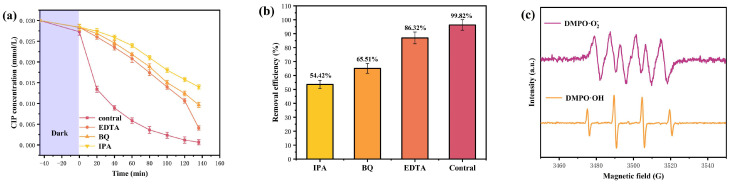
(**a**) The variation curves of CIP saturation concentration by different free radical scavengers, and (**b**) the effect of different free radical scavengers on RME of CIP during photocatalytic degradation process, and (**c**) EPR spectrum of the hydroxyl radical and the superoxide anion by using HBHP.

In order to investigate the influence of different radicals on the removal efficiency of CIP in wastewater under visible light irradiation conditions, this study added BQ, EDTA, and IPA as radical scavengers in the initial stage of the photocatalytic degradation experiment using HBHP. The saturation concentration of the scavengers (BQ, IPA, or EDTA) was 0.15 mmol/L, and the incremental volume was 1 mL. Figure 11a displays the variational curves of CIP saturation concentration using different free radical scavengers using HBHP. Figure 11b reveals the effect of different free radical scavengers on RME during the CIP degradation process using HBHP. It could be seen from Figure 11a,b that within the same irradiation time, the saturation concentration of CIP decreased with BQ, EDTA, or IPA as scavengers which appeared in the reaction systems, indicating that superoxide anion (•O_2_^−^), hydroxyl radical (•OH), and hole (h^+^) became the active radicals and participated in the degradation reaction of CIP. In accordance with Figure 11a and Figure 11b, compared with the control group, the average RME of CIP decreased by 45.58%, 34.49%, or 13.68% in the presence of IPA, BQ, or EDTA, respectively. It could be concluded from Figure 11a,b that among above active radicals, •OH showed the strongest oxidation ability and made the greatest contribution for degrading CIP. The oxidation removal ability of three active species was ranked as the following sequence: •OH > •O_2_^−^ > h^+^. The generation of active free radicals, specifically superoxide radicals (•O_2_^−^) and hydroxyl radicals (•OH), during the photocatalytic degradation process of CIP using HBHP, was studied by electron paramagnetic resonance (EPR) analysis [114]. Figure 11c displays the EPR spectrum during the photocatalytic degradation process of CIP using HBHP. As presented in Figure 11c, the characteristic EPR signals which corresponded to both •O_2_^−^ and •OH were clearly detected. According to Figure 11c, in the DMPO•O_2_^−^ adduct spectrum, four distinct peaks with an intensity ratio of 1:1:1:1 were observed; thus, the above results confirmed the presence of superoxide radicals. Concurrently, based on Figure 11c, the DMPO•OH adduct exhibited a typical quartet signal with an intensity ratio of 1:2:2:1, and as a result, strong evidence for the formation of hydroxyl radicals was gained. In a word, the above findings collectively demonstrated that both •O_2_^−^ radicals and •OH radicals were actively involved in the photocatalytic degradation process of CIP; simultaneously, •O_2_^−^ radicals and •OH radicals played significant roles in the oxidative decomposition of CIP.

### 3.4. Photocatalytic Mechanism Analysis

**Figure 12:**
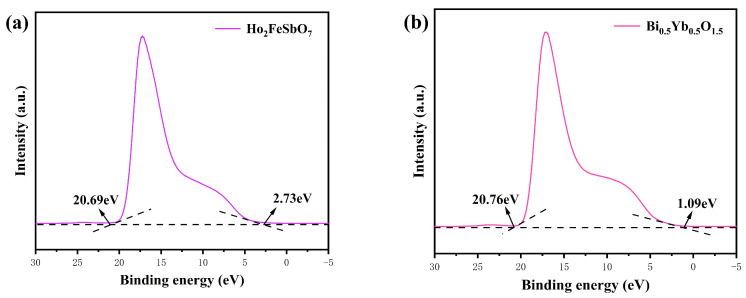
The UPS spectra of (**a**) Ho_2_FeSbO_7_ and (**b**) Bi_0.5_Yb_0.5_O_1.5_.

Figure 12 shows the ultraviolet photoelectron spectra (UPS) of Ho_2_FeSbO_7_ and Bi_0.5_Yb_0.5_O_1.5_. The ionization potential of the Ho_2_FeSbO_7_ catalyst or the Bi_0.5_Yb_0.5_O_1.5_ catalyst could be determined by a UPS test. In accordance with Figure 12, as to Ho_2_FeSbO_7_, the measured cutoff kinetic energies were 2.73 eV and 20.69 eV, respectively. Combining with the excitation energy (approximately 21.2 eV), the ionization potential of the valence band for Ho_2_FeSbO_7_ was calculated to be 3.24 eV according to Figure 12 [115]. Additionally, the corresponding measured cutoff kinetic energy values for Bi_0.5_Yb_0.5_O_1.5_ were 1.09 eV and 20.76 eV, respectively; as a result, the ionization potential of valence band for Bi_0.5_Yb_0.5_O_1.5_ was calculated to be 1.53 eV. The above results further indicated that the conduction band potential of Ho_2_FeSbO_7_ or Bi_0.5_Yb_0.5_O_1.5_ was 0.98 eV or −0.77 eV, respectively. These results provided strong support for the reaction mechanism model which was proposed in this study.

**Figure 13:**
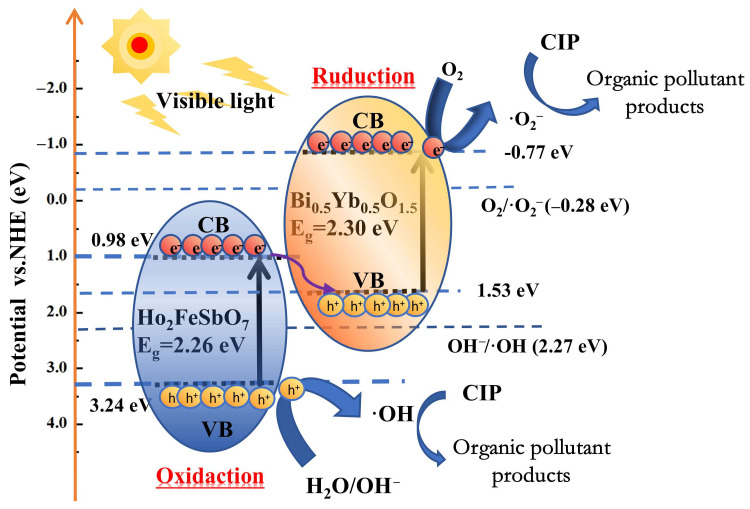
The mechanism diagram of CIP degradation by using HBHP.

Figure 13 shows the schematic diagram of the mechanism for the photocatalytic degradation of CIP by HBHP. It could be seen from Figure 13 that the valence band potential (*E_VB_* = 3.24 eV) of Ho_2_FeSbO_7_ was higher than that (*E_VB_* = 1.53 eV) of Bi_0.5_Yb_0.5_O_1.5_; thus, the photo-induced holes in the valence band of Ho_2_FeSbO_7_ tended to migrate to the valence band of Bi_0.5_Yb_0.5_O_1.5_. Correspondingly, the conduction band potential (*E_CB_* = −0.77 eV) of Bi_0.5_Yb_0.5_O_1.5_ was lower than that (*E_CB_* = 0.98 eV) of Ho_2_FeSbO_7_, indicating that the photo-induced electrons in the conduction band of Bi_0.5_Yb_0.5_O_1.5_ could migrate to the conduction band of Ho_2_FeSbO_7_. Due to the standard redox potential of OH^−^/•OH (2.27 eV vs. NHE, pH = 7) being higher than that of the E_VB_ for Bi_0.5_Yb_0.5_O_1.5_ (1.53 eV), it was impossible to oxidize OH^−^ for generating •OH radicals over the E_VB_ for Bi_0.5_Yb_0.5_O_1.5_ [116]. Similarly, the standard potential of O_2_/•O_2_^−^ (−0.28 eV vs. NHE, pH = 7) was lower than that of the *E_CB_* of Ho_2_FeSbO_7_ (0.98 eV); thus, the reduction in O_2_ for forming •O_2_^−^ could not be achieved either over the *E_CB_* of Ho_2_FeSbO_7_ [117]. However, as indicated by the EPR spectra and radical trapping experiments in Figure 11, the presence of •O_2_^−^ and •OH was indeed detected in the reaction system; thus, the above results contradicted the above energy level analysis results.

Previous studies have suggested that HBHP might belong to a Z-scheme heterojunction structure [118]. At the interface of HBHP, the energy band of Ho_2_FeSbO_7_ bent downward, while the energy band of Bi_0.5_Yb_0.5_O_1.5_ bent upward; thus, a built-in field was formed between the *E_CB_* (0.98 eV) of Ho_2_FeSbO_7_ and the *E_VB_* (1.53 eV) of Bi_0.5_Yb_0.5_O_1.5_. Under the drive of the built-in field, electrons on the conduction band of Ho_2_FeSbO_7_ flowed directly to the valence band of Bi_0.5_Yb_0.5_O_1.5_ and underwent recombination. This effectively concentrated strong oxidizing holes in the valence band of Ho_2_FeSbO_7_ and concentrated strong reducing electrons in the conduction band of Bi_0.5_Yb_0.5_O_1.5_. The conduction band potential (*E_CB_* = −0.77 eV) of Bi_0.5_Yb_0.5_O_1.5_ was sufficient to reduce O_2_ for forming •O_2_^−^; meanwhile, the valence band potential (*E_VB_* = 3.24 eV) of Ho_2_FeSbO_7_ possessed a high potential to oxidize OH^−^ for forming •OH radicals. Based on the above analysis, it could be confirmed that the HBHP system owned a Z-scheme heterojunction structure. The structure of HBHP helped to enhance the electron migration efficiency and effectively suppressed the recombination process of photogenerated carriers; thereby, the photocatalytic performance of HBHP was significantly improved.

In this study, a liquid chromatography–mass spectrometry (LC-MS) system was employed to conduct a systematic analysis of the intermediate products which were generated during the degradation of CIP by HBHP. A total of 10 different intermediates were identified.

**Figure 14:**
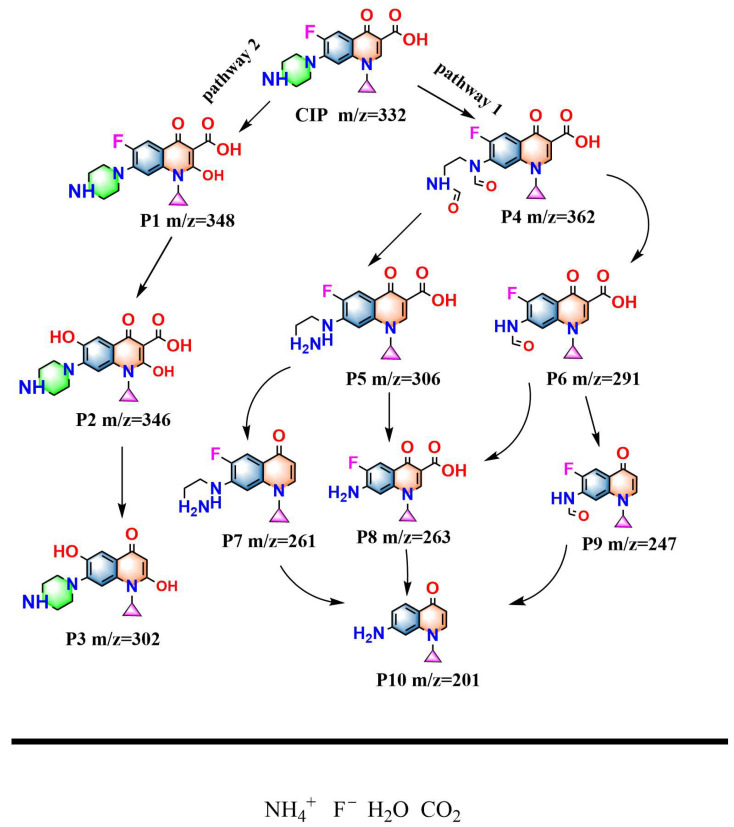
Possible degradation pathways of CIP by using HBHP.

Based on the detected intermediate products, the possible degradation pathways of CIP during the photocatalytic process were speculated. Figure 14 shows the possible degradation pathways of CIP using HBHP. It could be revealed from Figure 14 that the photocatalytic process of CIP mainly included the cleavage of the piperazine ring, defluorination (or decarboxylation) of the quinolone nucleus, hydroxylation, and ring-opening reactions; ultimately, the above intermediate products were mineralized into inorganic ions such as CO_2_, H_2_O, F^−^, and NH_4_^+^. As shown in Figure 14, under the condition of visible light irradiation, active radicals such as •OH and •O_2_^−^ were generated on the surface of the catalysts, attacking the CIP molecules. The piperazine ring which was contained within the CIP molecule was the most vulnerable site which could be easily attacked by •O_2_^−^ and •OH and underwent cleavage during the photocatalytic reactions. For instance, in Pathway 1, the piperazine ring first underwent oxidative ring-opening for forming the intermediate product P4 (C_17_H_16_FN_3_O_5_, *m*/*z* = 362) [119]. Notably, P4 could further be degraded in two different directions: one direction was by means of decarboxylation for generating P5 (C_15_H_16_FN_3_O_3_, *m*/*z* = 306), which was desethylciprofloxacin with significantly reduced antibacterial activity; simultaneously, another direction was by means of decarboxylation and further cleavage of the piperazine ring for producing P6 (C_15_H_16_FN_3_O_4_, *m*/*z* = 291) [120,121].

In addition, the study found that P5 intermediate and P6 intermediate could generate the same product 7-aminoquinolone P8 (C_13_H_11_FN_2_O_3_, *m*/*z* = 263) in the subsequent reactions [122]. Subsequently, the C-F bond and carboxyl group on the quinolone nucleus of P8 were prone to be attacked, and subsequently, defluorination reaction and decarboxylation reactions occurred for forming P10 (C_12_H_12_N_2_O, *m*/*z* = 201); ultimately, the F^−^ ions were released [123]. Further research indicated that P10 underwent a ring-opening reaction of the quinolone ring and was gradually oxidized and decomposed into small molecular substances such as oxalic acid (C_2_H_2_O_4_), formic acid (CH_2_O_2_), and CO_2_; finally, the complete mineralization of CIP was realized. In addition, the study also found that desethylciprofloxacin (P5) could first undergo decarboxylation for form P7 (C_14_H_16_N_3_O, *m*/*z* = 261); subsequently, the defluorination reaction and piperazine ring cleavage reaction occurred for generated P10 [123], while P6 could be decarboxylated for forming P9 (C_13_H_11_FN_2_O_2_, *m*/*z* = 247), and then the defluorination reaction and the decarbonylation reaction also occurred for generating P10. The above intermediate products were ultimately further mineralized into CO_2_ and H_2_O; contemporaneously, the effective degradation of CIP was realized.

Furthermore, this study also proposed another possible degradation, Pathway 2. In Pathway 2, hydroxylation occurred at the C8 site of the quinolone nucleus of CIP; subsequently, the intermediate product P1 (8-hydroxy ciprofloxacin, C_17_H_18_FN_3_O_4_, *m*/*z* = 348) was generated [124]. Then, the C-F bond in the quinolone nucleus broke, forming P2 (C_17_H_19_N_3_O_5_, *m*/*z* = 346). Subsequently, a further decarboxylation reaction occurred, generating P3 (C_16_H_19_N_3_O_3_, *m*/*z* = 302) [125,126]. P3 further underwent a ring-opening reaction and oxidation reaction under the attack of free radicals; eventually, the above intermediate products were mineralized into CO_2_ and H_2_O.

In conclusion, this study conducted a systematic analysis of the reaction pathways of CIP during the photocatalytic degradation process using HBHP and proposed two main degradation mechanisms. By means of the analysis of the multi-path degradation behavior of CIP, we offered a theoretical basis and technical support for the construction of an efficient and low-risk targeted catalytic system.

## 4. Conclusions

In summary, this study successfully synthesized the pyrochlore-type structure photocatalyst Ho_2_FeSbO_7_ and the fluorite-type structured photocatalyst Bi_0.5_Yb_0.5_O_1.5_. A series of characterization techniques including XRD, FTIR, Raman spectroscopy, UV-vis diffuse reflectance spectroscopy, XPS, UPS, TEM-EDS mapping, and EPR were employed to systematically investigate the microstructural features, elemental composition, and photoelectrochemical properties of the above photocatalysts. Furthermore, HBHP was successfully fabricated via a solvothermal approach. The degradation experiments demonstrated that HBHP exhibited outstanding performance for the visible-light-driven removal of CIP, as a result, the RME of 99.82% for CIP concentration and a RME of 97.63% for TOC saturation concentration were obtained after visible light irradiation of 140 min. The comparative analysis revealed that the photocatalytic activity of HBHP was 1.16 times, 1.36 times, or 2.52 times higher than that of Ho_2_FeSbO_7_, Bi_0.5_Yb_0.5_O_1.5_, or N-doped TiO_2_, respectively. The significant enhancement in catalytic performance could be attributed to the effective heterojunction photocatalyst which was formed by Ho_2_FeSbO_7_ and Bi_0.5_Yb_0.5_O_1.5_. HBHP facilitated efficient spatial separation and rapid migration of photogenerated charge carriers; thereby, the recombination of the photo-induced electrons and the photo-induced holes was suppressed; concurrently, the generation efficiency of the reactive species was enhanced. Radical trapping experiments and EPR analysis confirmed that hydroxyl radicals (•OH) were the dominant active species during the photocatalytic degradation process of CIP, while superoxide radicals (•O_2_^−^) and photogenerated holes (h^+^) also played contributing roles. Based on the identification and analysis of intermediate products, a plausible photocatalytic degradation pathway for CIP was proposed in this study.

In conclusion, this work successfully synthesized the Z-scheme heterojunction composite photocatalyst HBHP, and gained a deep understanding of the structural activity relationship of HBHP and the mechanism of photocatalytic reaction. The research results indicated that HBHP possessed broad application prospects for treating CIP-contaminated wastewater. Based on this study, further development of high-performance photocatalytic nanomaterials could be achieved by methods such as interface engineering, carrier dynamics regulation, and material active site enhancement. This study provided valuable theoretical guidance and experimental support for other researchers on studying how to optimize and design future advanced photocatalysts.

## Data Availability

Data are contained within the article.

## Supplementary Materials

The following supporting information can be downloaded at: https://www.mdpi.com/article/10.3390/nano15161290/s1, Figure S1: XRD pattern of Ho_2_FeSbO_7_; Figure S2: XRD pattern of Bi_0.5_Yb_0.5_O_1.5_; Figure S3: (a) XRD spectrum and Rietveld refinement of Ho_2_FeSbO_7_; (b) Crystal structure of Ho_2_FeSbO_7_; Figure S4: (a) XRD spectrum and Rietveld refinement of Bi_0.5_Yb_0.5_O_1.5_; (b) Crystal structure of Bi_0.5_Yb_0.5_O_1.5_; Figure S5: EDS spectrum of HBHP; Figure S6: XRD spectrum of N-doped TiO_2_; Figure S7: The UV-Vis diffuse reflectance spectra of N-doped TiO_2_; Figure S8: XRD pattern of HBHP after the cyclic degradation experiment; Figure S9: SEM image of HBHP after the cyclic degradation experiment; Figure S10: The influence of different anions on the degradation removal of CIP by using HBHP under visible light irradiation; Figure S11: The influence of different anions on the degradation efficiency of CIP by using HBHP under visible light irradiation; Figure S12: The influence of different pH values on the photocatalytic degradation efficiency of CIP by using HBHP under the condition of visible light irradiation; Figure S13: The effect of initial CIP concentration on the photocatalytic degradation efficiency of CIP by using HBHP under the condition of visible light irradiation; Table S1: Atomic characteristic values of the crystal structure of Ho_2_FeSbO_7_; Table S2: Atomic characteristic values of the crystal structure of Bi_0.5_Yb_0.5_O_1.5_; Table S3: The impact of different catalyst amount of HBHP on the removal efficiency of CIP.
