# Supplementary material for: The Synthesis and Photophysical Performance of a Novel Z-Scheme Ho2FeSbO7/Bi0.5Yb0.5O1.5 Heterojunction Photocatalyst and the Photocatalytic Degradation of Ciprofloxacin Under Visible Light Irradiation
Source: Nanomaterials (Basel). 2025 Aug 21;15(16):1290. doi: 10.3390/nano15161290 (PMC12389380; doi:10.3390/nano15161290)
Supplement: Supplementary file 1 [file nanomaterials-15-01290-s001.zip › nanomaterials-3780590-supplementary.pdf]

nanomaterials

Supplementary Information

The Synthesis and Photophysical Performance of a Novel Z-Scheme $Ho_2FeSbO_7/Bi_{0.5}Yb_{0.5}O_{1.5}$ Heterojunction Photocatalyst and the Photocatalytic Degradation of Ciprofloxacin Under Visible Light Irradiation

Jingfei Luan [1,2,*], Anan Liu [1], Liang Hao [1], Boyang Liu [1] and Hengchang Zeng [1]

[1] School of physics, Changchun Normal University, Changchun 130032, China; ananliu2001@outlook.com (A.L.); 19845486007@139.com (L.H.); boyangliu152@outlook.com (B.L.); zenghc23@mails.jlu.edu.cn (H.Z.)
[2] State Key Laboratory of Pollution Control and Resource Reuse, School of the Environment, Nanjing University, Nanjing 210093, China
* Correspondence: jfluan@nju.edu.cn; Tel.: +86-199-5193-9498

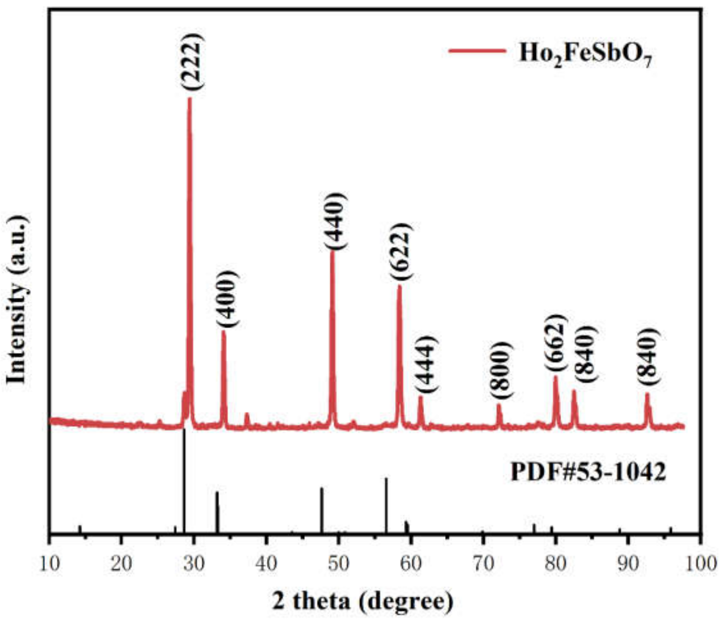

Figure S1. XRD pattern of Ho_2FeSbO_7.

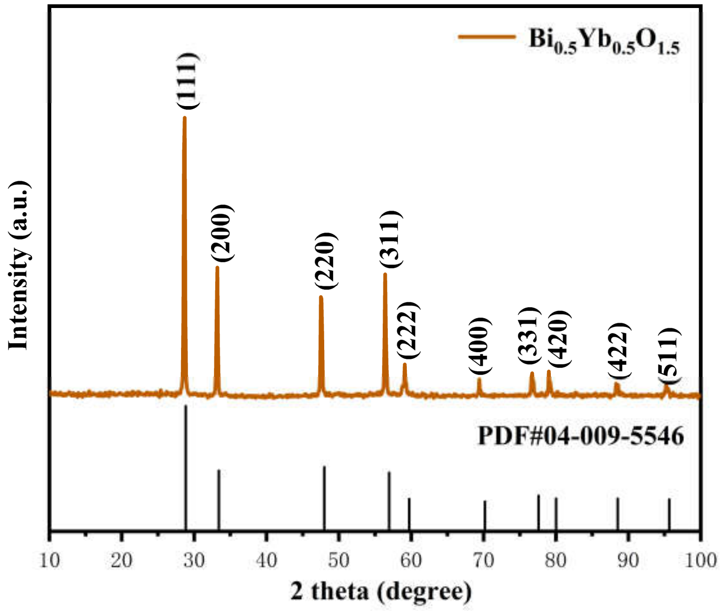

Figure S2. XRD pattern of $Bi_{0.5}Yb_{0.5}O_{1.5}$.

Table S1. Atomic characteristic values of the crystal structure of Ho_2FeSbO_7.

**Atom**	**positions**	**x**	**y**	**z**	**Occupation Factor**
**Ho**	**16d**	**0.5**	**0.5**	**0.5**	**1**
**Fe**	**16c**	**0**	**0**	**0**	**0.5**
**Sb**	**16c**	**0**	**0**	**0**	**0.5**
**O(1)**	**48f**	**0.4286**	**0.125**	**0.125**	**1**
**O(2)**	**8b**	**0.375**	**0.375**	**0.375**	**1**

Table S2. Atomic characteristic values of the crystal structure of $Bi_{0.5}Yb_{0.5}O_{1.5}$.

**Atom**	**positions**	**x**	**y**	**z**	**Occupation Factor**
**Bi**	**4a**	**0**	**0**	**0**	**0.5**
**Yb**	**4a**	**0**	**0**	**0**	**0.5**
**O**	**8c**	**0.25**	**0.25**	**0.25**	**0.75**

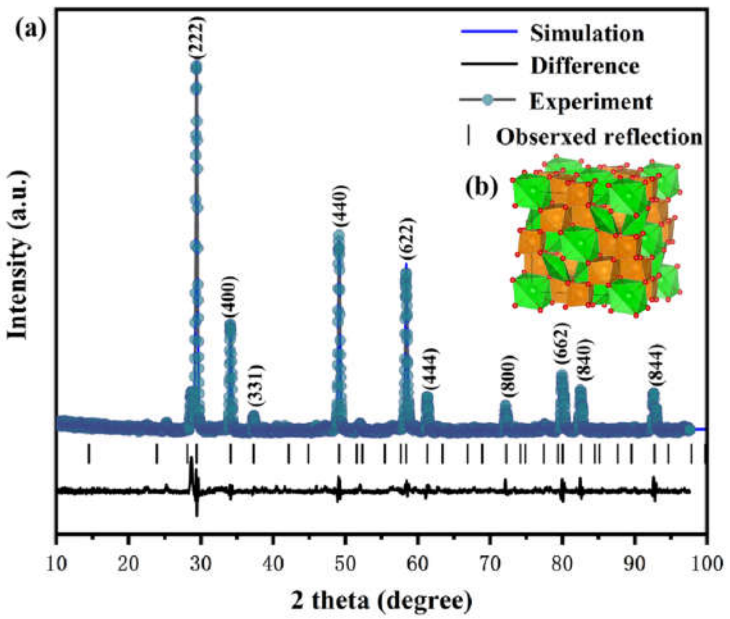

Figure S3. (a) XRD spectrum and Rietveld refinement of Ho_2FeSbO_7; (b) Crystal structure of Ho_2FeSbO_7.

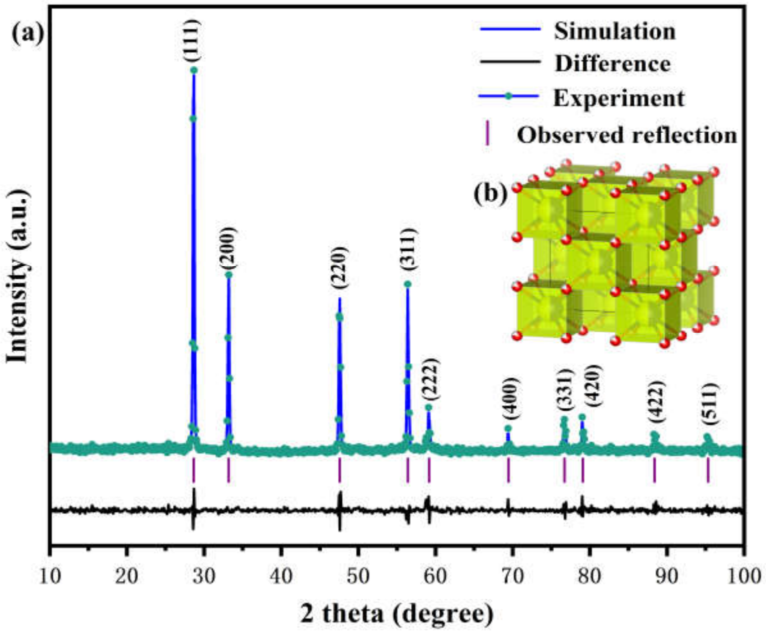

Figure S4. (a) XRD spectrum and Rietveld refinement of $Bi_{0.5}Yb_{0.5}O_{1.5}$; (b) Crystal structure of $Bi_{0.5}Yb_{0.5}O_{1.5}$.

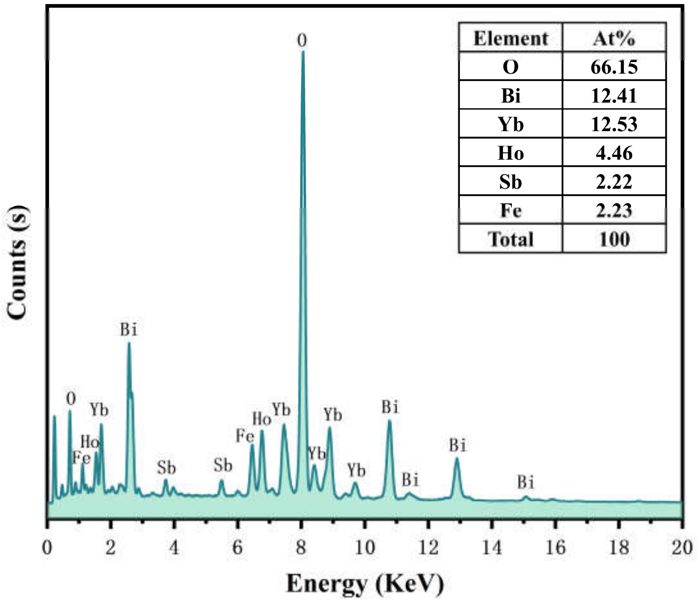

Figure S5. EDS spectrum of HBHP

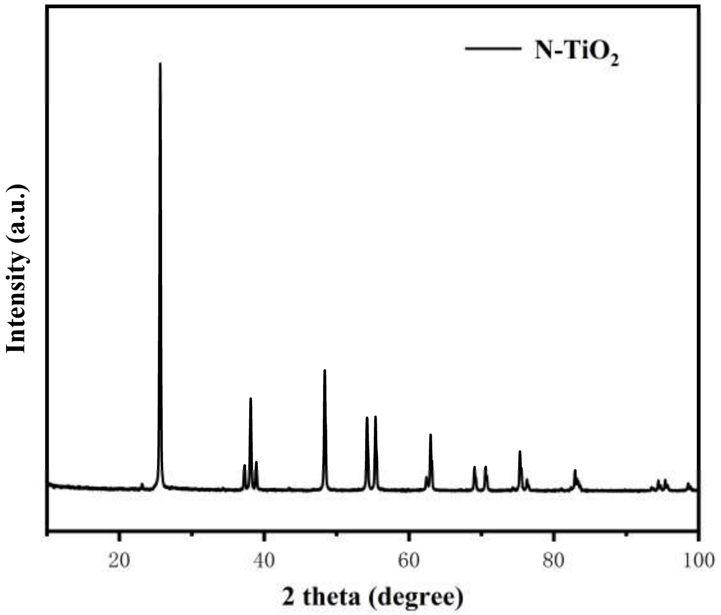

Figure S6. XRD spectrum of N-doped TiO_2.

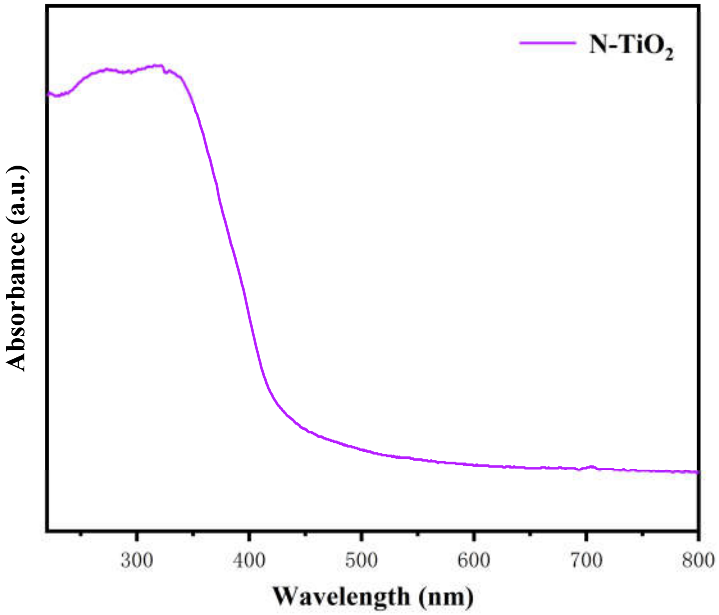

Figure S7. The UV-Vis diffuse reflectance spectra of N-doped TiO_2.

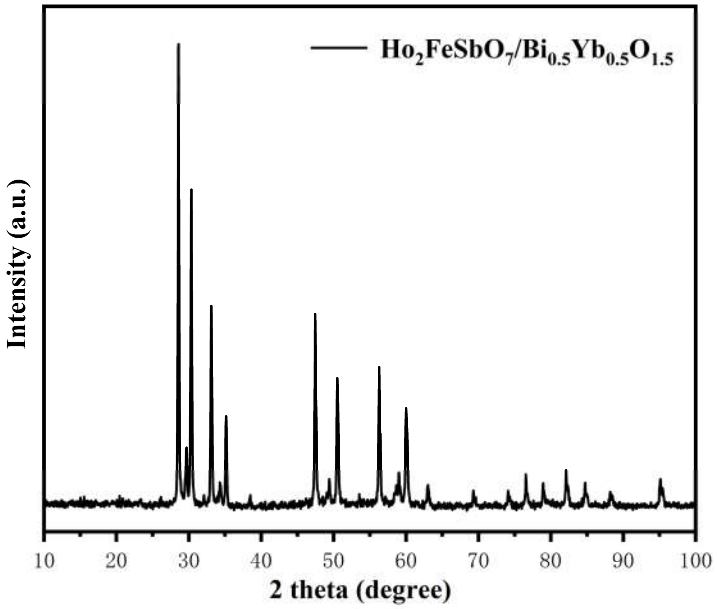

Figure S8. XRD pattern of HBHP after the cyclic degradation experiment.

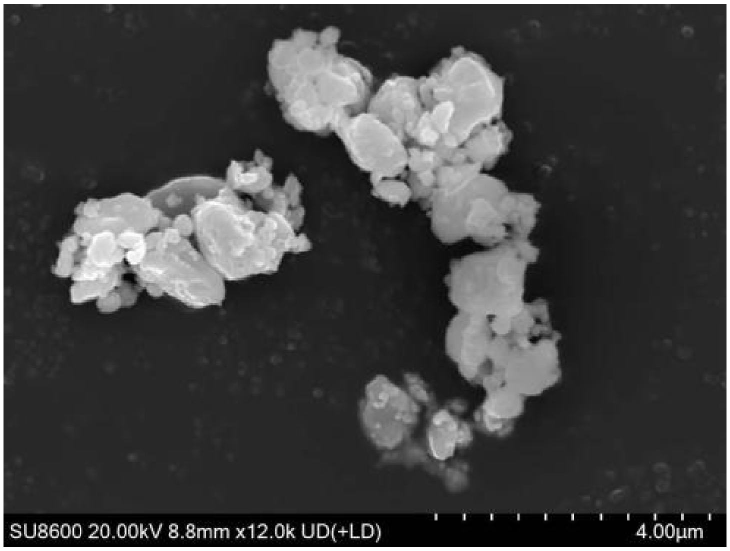

Figure S9. SEM image of HBHP after the cyclic degradation experiment.

.

Table S3. The impact of different catalyst amount of HBHP on the removal efficiency of CIP.

Catalyst amount (g/L)	Removal efficiency (%)	Unit usage efficiency (mg/g·h)
0.05	68%	36.0
0.10	92%	92.0
0.12	98%	98.2
0.15	94%	62.7
0.20	95%	47.5

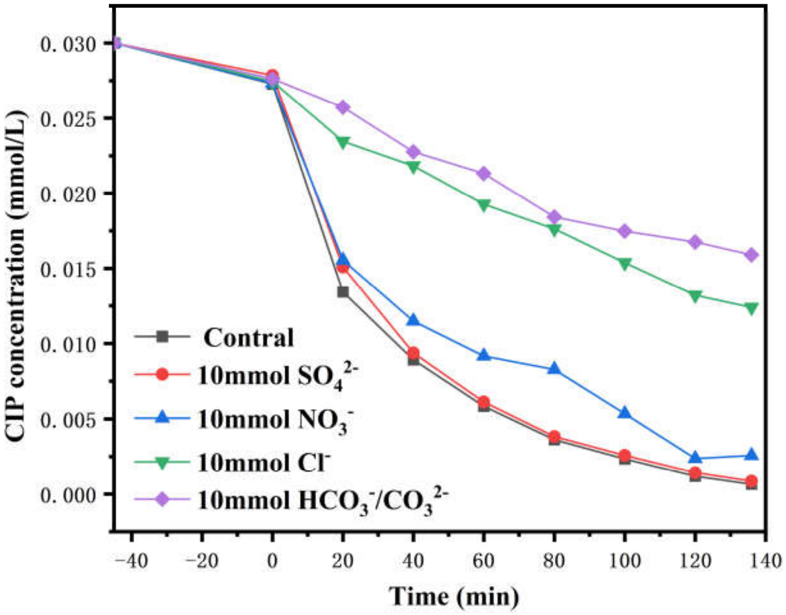

Figure S10. The influence of different anions on the degradation removal of CIP by using HBHP under visible light irradiation.

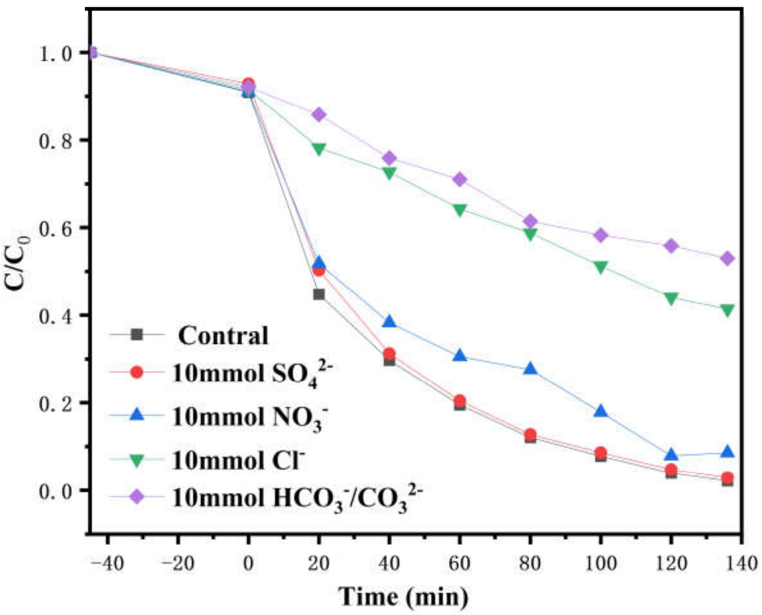

Figure S11. The influence of different anions on the degradation efficiency of CIP by using HBHP under visible light irradiation.

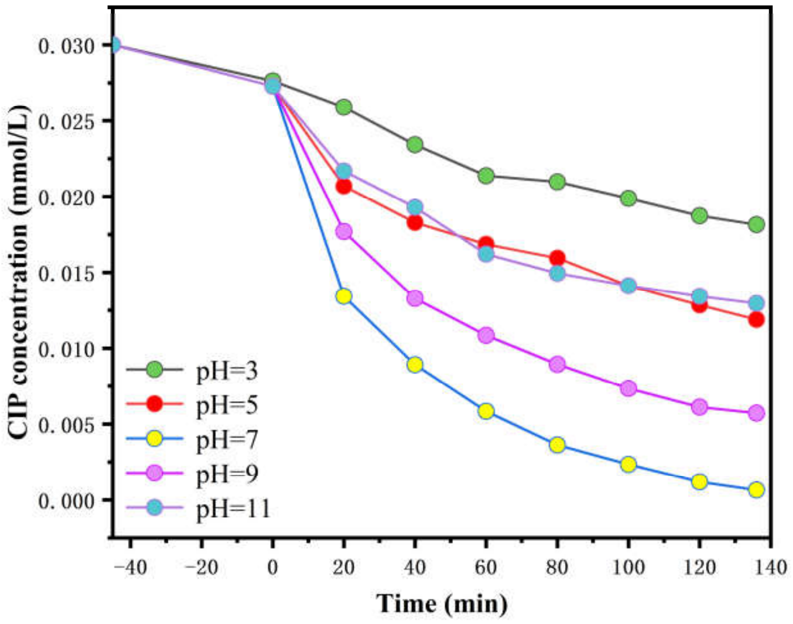

Figure S12. The influence of different pH values on the photocatalytic degradation efficiency of CIP by using HBHP under the condition of visible light irradiation.

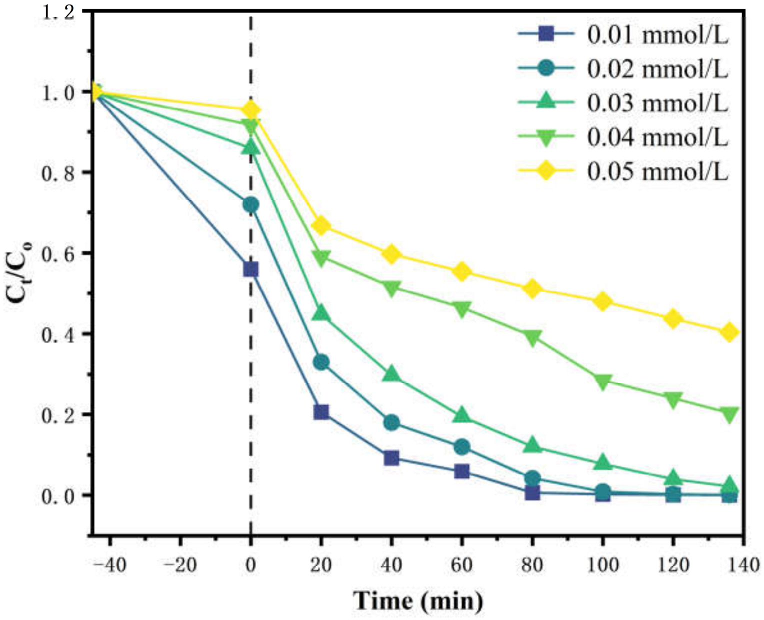

Figure S13. The effect of initial CIP concentration on the photocatalytic degradation efficiency of CIP by using HBHP under the condition of visible light irradiation.
